# Multiple sex chromosome systems in howler monkeys (Platyrrhini, Alouatta)

**DOI:** 10.3897/CompCytogen.v8i1.6716

**Published:** 2014-02-25

**Authors:** Eliana Ruth Steinberg, Mariela Nieves, Marta Dolores Mudry

**Affiliations:** 1 Grupo de Investigación en Biología Evolutiva (GIBE) – Departamento de Ecología, Genética y Evolución – Facultad de Ciencias Exactas y Naturales – Universidad de Buenos Aires – IEGEBA (CONICET-UBA) – Ciudad Universitaria – Pab. II –4° piso - Labs 43-46 - (C1428EGA) – Buenos Aires – Argentina

**Keywords:** Multiple sex chromosome systems, combined phylogenetic analysis, FISH, Neotropical Primates, cytochrome b

## Abstract

In light of the multiple sex chromosome systems observed in howler monkeys (*Alouatta* Lacépède, 1799) a combined cladistic analysis using chromosomal and molecular characters was applied to discuss the possible origin of these systems. Mesoamerican and South American howlers were karyologically compared. FISH analysis using the chromosome painting probes for the #3 and #15 human chromosomes was applied to corroborate the homeology of the sexual systems. We found that the HSA3/15 syntenic association, present in the sex chromosome systems of South American Howlers, is not present in those of Mesoamerican ones. The autosomes involved in the translocation that formed the sexual systems in the Mesoamerican and South American species are different, thus suggesting an independent origin. Parsimony analysis resolved the phylogenetic relationships among howler species, demonstrating utility of the combined approach. A hypothesis for the origin of the multiple sex chromosome systems for the genus is proposed.

## Introduction

Howler monkeys (genus *Alouatta* Lacépède, 1799 of the family Atelidae) exhibit one of the widest geographic distributions recorded to date for Neotropical Primates. Their distribution extends from southern Mexico to northern Argentina (Crockett and Eisenberg 1987, Rylands 2000). They inhabit a diverse range of environments, including tropical rain forests, flood forests, gallery forests, patches of forest and deciduous and semideciduous seasonal environments (Crockett and Eisenberg 1987, Zunino et al. 2001). There remains a lack of consensus regarding both the number of species within the genus, which, depending on the author, ranges from 9 to 14 species (Rylands 2000, Groves 2001, 2005, Gregorin 2006, Rylands and Mittermeier 2009), and the phylogenetic relationships among them. This shows the complexity of the taxonomy of *Alouatta* and highlights the importance of including a larger number of variables for a more accurate characterization of the species in the genus. We adhere to the classification proposed by Groves (2001, 2005) in recognizing 10 species (*Alouatta belzebul* Linnaeus, 1766, *Alouatta seniculus* Linnaeus, 1766, *Alouatta sara* Elliot, 1910, *Alouatta macconnelli* Linnaeus, 1766, *Alouatta caraya* Humboldt, 1812, *Alouatta palliata* Gray, 1849, *Alouatta pigra* Lawrence, 1933, *Alouatta guariba* Humboldt, 1812, *Alouatta nigerrima* Lönnberg, 1941, *Alouatta coibensis* Thomas, 1912), since it considers both morphological and genetic information.

In this context, and to contribute to the description of the phylogenetic relationships in the genus, several authors have proposed that chromosomal data can also be used as phylogenetic markers, since they are inherited as mendelian characters and are conserved within species (Sankoff 2003, Dobigny et al. 2004, Stanyon et al. 2008). Following the Maximum Parsimony criterion, karyological comparisons allow the identification of chromosomal forms shared by common ancestrality.

In primates, different researchers in the last three decades have proposed chromosomal speciation as a probable evolutionary mechanism to explain the diversity observed in living species (de Grouchy et al. 1972, Seuánez 1979, Dutrillaux and Couturier 1981, Clemente et al. 1990, Stanyon et al. 2008, de Oliveira et al. 2012). In howler monkeys, species exhibit diploid numbers (2N) ranging from 44 in *Alouatta seniculus* to 58 in *Alouatta pigra*, and in a large number of species, multiple sex chromosome systems in males originated from Y-autosome translocations have been described (Table 1). The chromosomes involved in the Y-autosome translocations in *Alouatta caraya*, *Alouatta macconnelli*, *Alouatta guariba guariba* Humboldt, 1812, *Alouatta guariba clamitans* Cabrera, 1940, *Alouatta sara* and *Alouatta seniculus arctoidea* Cabrera, 1940, are homeologous to the same regions of human chromosomes #3 and #15 (Consigliere et al. 1996, 1998, Mudry et al. 2001, de Oliveira et al. 2002).

**Table 1.** Cytogenetic characteristics of howler monkeys (*Alouatta*).

Species	2N	Sex Chromosome Systems	References
*Alouatta belzebul*	♀50 ♂49	X_1_X_1_X_2_X_2_/X_1_X_2_Y	Armada et al. 1987^§^
*Alouatta seniculus seniculus*	♀♂47 to 49†	XY	Yunis et al. 1976
*Alouatta seniculus stramineus*	♀♂47 to 49†	X_1_X_1_X_2_X_2_/X_1_X_2_Y_1_Y_2_	Lima and Seuánez 1991^§^
*Alouatta seniculus arctoidea*	♀44 ♂45 ‡	X_1_X_1_X_2_X_2_/X_1_X_2_Y_1_Y_2_	Stanyon et al. 1995
*Alouatta sara*	♀♂ 48 to 51†	X_1_X_1_X_2_X_2_/X_1_X_2_Y	Minezawa et al. 1985
	♀♂50	X_1_X_1_X_2_X_2_/X_1_X_2_Y_1_Y_2_	Stanyon et al. 1995
*Alouatta macconnelli*	♀♂ 47 to 49†	X_1_X_1_X_2_X_2_/X_1_X_2_Y_1_Y_2_	Lima et al. 1990
*Alouatta caraya*	♀♂52	XX/XY	Egozcue and De Egozcue 1966, Mudry et al. 1984, 1994
		X_1_X_1_X_2_X_2_/X_1_X_2_Y_1_Y_2_	Rahn et al. 1996^§^, Mudry et al. 1998^§^, 2001^§^
*Alouatta palliata*	♀♂56	XX/XY	Torres and Ramírez 2003
	♀54 ♂53	X_1_X_1_X_2_X_2_/X_1_X_2_Y	Ma et al. 1976, Solari and Rahn 2005^§^
*Alouatta pigra*	♀♂58	X_1_X_1_X_2_X_2_/X_1_X_2_Y_1_Y_2_	Steinberg et al. 2008^§^
*Alouatta guariba guariba*	♀50 ♂49	XX/XY	Koiffmann and Saldanha 1974
	♂49	X_1_X_2_Y	de Oliveira et al. 1995
	♀50♂49	X_1_X_1_X_2_X_2_X_3_X_3_/X_1_X_2_X_3_Y_1_Y_2_	de Oliveira et al. 2002
*Alouatta guariba clamitans*	♀46 ♂45	XX/XY	de Oliveira et al. 1995
		X_1_X_1_X_2_X_2_/X_1_X_2_Y	de Oliveira et al. 1998
		X_1_X_1_X_2_X_2_X_3_X_3_/X_1_X_2_X_3_Y_1_Y_2_	de Oliveira et al. 2002
*Alouatta nigerrima*	♀50	XX	Armada et al. 1987
*Alouatta coibensis*	ND	ND	---

† These differences are due to the presence of microchromosomes (1 to 3 per nuclei);

‡ Differences due to a variation in microchromosome number between sexes. ND: not yet cytogenetically characterized.

^§^ Meiotic studies performed to corroborate the sex chromosome system.

The phylogenies proposed so far for *Alouatta* have used either molecular markers (γ^1^-globin (Meireles et al. 1999), Mt ATP synt 8 and 6, Mt cyt b, CAL and RAG1 (Cortés-Ortiz et al. 2003)) or chromosomal characters (de Oliveira et al. 2002). However, the combination of different variables can improve the phylogenetic signal due to the possible common shared history of different datasets. At the same time, this combination can increase the support of a tree, since different characters evolve at particular rates and will support different parts of the tree (Kluge 1989, Whittaker et al. 2007).

In the present contribution, howler species were karyologically compared and FISH analyses were carried out to corroborate the homeology of the sex chromosome systems among them. Using these data and molecular data obtained from the literature, a phylogenetic analysis combining them in a single matrix was performed.

## Methods

*Sampled specimens*: A total of 29 adult specimens of both sexes of four species of howlers, both from captivity as well as from the wild within their natural geographical distribution, were analyzed: *Alouatta caraya* (9 ♂ and 6 ♀), *Alouatta guariba clamitans* (1 ♂), *Alouatta pigra* (6 ♂ and 5 ♀) and *Alouatta palliata* (2 ♂).

The origin of the animals was as follows:

**Argentina**

*Alouatta caraya*, 1 ♂ from Corrientes Zoo, Corrientes; 1 ♂ from Ecological Park “El Puma”, Misiones; 1 ♂ and 2♀ from Mendoza Zoo, Mendoza; 6 ♂ and 4 ♀ from the Black Howler Monkey Reeducational Center, La Cumbre, Córdoba.

*Alouatta guariba clamitans*, 1 ♂ from “Güira-Oga”, Misiones.

**Mexico**

*Alouatta pigra*: 4 ♂ and 4 ♀ were sampled in the wild in Campeche, Yucatán Península; 2 ♂ and 1 ♀ from San Juan de Aragón Zoo, Mexico City.

*Alouatta palliata*: 1 ♂ from San Juan de Aragón Zoo, Mexico City; 1 ♂ from Chapultepec Zoo, Mexico City.

### Classical cytogenetic analysis

*Chromosome preparation*: Peripheral blood samples were collected from all animals with previously heparinized disposable syringes. Lymphocytes were cultured for 72 h at 37 °C following Mudry (1990). At least 50 metaphases were analyzed to determine the diploid number (2N) at 1000×. Metaphase spreads were treated with G-Wright banding (Steinberg et al. 2007). At least 10 G-banded metaphases with the species diploid number (2N) were photographed with a Leica DFC 340 FX camera. Chromosomes were arranged according to previously described karyotypes using Photoshop CS (Adobe) and the species assignation of each specimen was corroborated.

*Analysis of homeologies*: For *Alouatta caraya* and *Alouatta guariba clamitans*, the homeologies with human chromosomes and the homeologies with the other South American howlers are well known (Consigliere et al. 1998, Mudry et al. 2001, de Oliveira et al. 2002, Stanyon et al. 2011). The G-banded chromosomes of *Alouatta pigra* and *Alouatta palliata* were first compared with those of *Alouatta caraya* and *Alouatta guariba clamitans*. We took *Alouatta caraya*’s karyotype as the reference for the comparisons with Mesoamerican howlers (Mudry et al. 2001, Szapkievich and Mudry 2003). To compare homeologies, the G-banded metaphases obtained for *Alouatta caraya*, *Alouatta guariba clamitans*, *Alouatta pigra* and *Alouatta palliata* were also compared with those published for *Alouatta guariba guariba* (de Oliveira et al. 2002, Stanyon et al. 2011), *Alouatta macconnelli* (de Oliveira et al. 2002), *Alouatta seniculus arctoidea* (Consigliere et al. 1996), *Alouatta belzebul* (Armada et al. 1987, Consigliere et al. 1998) and *Alouatta sara* (Consigliere et al. 1996).

### Cytomolecular study

FISH analysis with human chromosome painting probes #3 and #15 was used as a tool to confirm the identity of the sex chromosome systems in howlers. Whole chromosome painting probes for human chromosomes #3 (red), #15 (green), #21 (green), X (green) and Y (red) (PCT3 Cy3, PCT15 FITC, PCT21 FITC, PCTX FITC, PCTY Cy3, LEXEL S.R.L., Buenos Aires, Argentina) were used for FISH analysis on the metaphases of *Alouatta pigra*, *Alouatta caraya*, *Alouatta guariba clamitans* and *Alouatta palliata*. *Homo sapiens* (HSA) metaphases were used as a positive control of hybridization. The HSA3/21 syntenic association, considered ancestral in mammals and conserved in most primate species (Müller et al. 2000), was analyzed simultaneously as a control of synteny conservation. Human X and Y chromosomes were also tested.

FISH was performed according to the supplier’s instructions (LEXEL S.R.L., Buenos Aires, Argentina). Slides were counterstained with DAPI (Sigma) and analyzed with a Leica DMLB fluorescence microscope. Chromosome images were obtained with a Leica DFC 340 FX camera. Images were processed with Image Pro-Plus 4.5 (Media Cybernetics Inc.).

Our results were compared with those previously described (Consigliere et al. 1996, 1998, Mudry et al. 2001, de Oliveira et al. 2002, Stanyon et al. 2011).

### Phylogenetic analysis

*Chromosomal dataset*: We used data obtained from the comparisons of G-banding patterns and the analysis of chromosomal syntenic associations, both from the present study and from previous reports (Consigliere et al. 1996, 1998, García et al. 2001, 2002, Mudry et al. 2001, de Oliveira et al. 2002, Amaral et al. 2008, Stanyon et al. 2001, 2011). We considered the structural changes as characters. The pattern observed before and after their occurrence, i.e. their presence or absence, was considered as the character states. The matrix (see Appendix 1) was produced taking into consideration the characters proposed by Neusser et al. (2001) and modified for howlers by de Oliveira et al. (2002). These authors used an abbreviated nomenclature for ancestral Platyrrhini chromosome forms with a correspondence in the human karyotype. In the present contribution, for the character nomenclature, we refer directly to the human G band ideogram (Table 2). New characters were obtained from our karyological comparisons and introduced in the chromosomal dataset.

**Table 2.** Human chromosome syntenic association considered as characters and used to construct the binary matrix of chromosomal homeologies among howler monkeys (modified from Neusser et al. 2001, de Oliveira et al. 2002).

1. 1p21-pter/1p12-21 2. 5q31.3-qter/ 7p22; q11 q21 3. 5pter-q31.2/5q31.3-qter 4. 2pter q12/16q 5. 4q31.3-qter/4q23-q31.2 6. 4q23-q31.2/4pter-q22 7. (10q/16p)_3_ 8. 6 9. 8p/18 10. 15q21.3-q24/15q13-q21.2 11. 15q11 q13; q25 qter 12. 7p21 p11; q11 q21; q22 qter 13. 8q 14. 12 15. 11 16. 13 17. 9 18. 3pter p24; p21 p12; q12 q13; q27 qter 19. 3p24 q21; q13 q26 20. 1q32 qter 21. 1q21 q31 22. 3p12/21 23. 10p 24. 22 25. X 26. Y 27. 5pter-q31.2 28. (5q31.3-qter/7p22; q11 q21)_2_ 29. 1p21-pter 30. 1p12-21 31. 4q31.3-qter 32. 4q23-q31.2/15q13-q212 33. 4pter-q22 34. 14/15q21.3-q24 35. (10q/16p)_2_/(10q/16p)_1_	36. 15q11 q13; q25 qter/Y 37. 2pter q12 38. 16q 39. 3p24 q21; q13 q26/ 15q11 q13; q25 qter 40. 11/5pter-q31.2 41. 5pter-q31.2/7p22; q11; q21 42. 12/9 43. 1p21-pter/2pter q12 44. 16q/4pter-q22 45. 22/14 46. 2q13 qter/20 47. 2q13 qter/4q23-q31.2 48. 8q/2q13 qter 49. 7p22; q11; q21/8q 50. 7p22; q11; q21/8q 51. 17/2pter q12/12 52. 2pter q12/12 53. 1q32 qter/11 54. 1q32 qter/(11/5pter-q31.2)_2_ 55. (11/5pter-q31.2)_2_ 56. 18/14 57. 3pter p24; p21 p12; q12 q13; q27 qter/15q21.3-q24 58. 3pter p24; p21 p12; q12 q13; q27 qter/15q21.3-q24/16q 59. 15q21.3-q24/16q 60. 17/10p 61. 17/10p/19 62. 10p/19 63. 22/20 64. 22/20/1q21 q31 65. 20/1q21 q31 66. (11/5pter-q31.2)_3_ 67. 1p12-21/8p 68. 7p21 p11; q11 q21; q22 qter /14/15q21.3-q24	69. (10q/16p)_2_ 70. 11/(10q/16p)_2_ 71. 10q/16p 72. 10q/16p/4pter-q22 73. 10p/10q/16p/4pter-q22 74. 10p/10q/16p 75. 19/13 76. 19/22 77. 22/1p21-pter 78. 19/22/1p21-pter 79. 2q13 qter/4q23-q31.2 80. 6/1p12-21 81. Y/15q11 q13; q25 qter/ 3p24 q21; q13 q26 82. 1p12-21/5pter-q31.2/7p22; q11; q21/5q31.3-qter/ 7p22; q11 q21 83. 9/22 84. 17/11 85. 3pter p24; p21 p12; q12 q13; q27 qter/8p 86. 15q21.3-q24/1q32 qter 87. 16q/15q21.3-q24/1q32 qter 88. 4pter-q22/1p12-21 89. 14 90. 2pter q12/4pter-q22 91. 15q13-q21.2/7p22; q11; q21/5q31.3-qter/ 7p22; q11; q21 92. 6/15q21.3-q24 93. 14/1p12-21 94. 6/15q21.3-q24/14/1p12-21 95. 17/8p/18 96. 22/10q/16p 97. 2q13 qter/11 98. (10q/16p)_2_/1q21 q31/20 99. Y/7

/: separates the chromosomal segments that constitute an association. ()_n_: n = number of repeats in the segment

*Molecular dataset*: The sequences available in GenBank for the same species used in the G-banding pattern and FISH comparisons were taken into to choose the molecular marker. The only molecular marker that fullfiled all the requirements was cyt b. The sequences used were (Genbank Accession Numbers): *Alouatta belzebul* (AY374348.2), *Alouatta caraya* (AY374359.2), *Alouatta seniculus arctoidea* (AY065886.1), *Alouatta sara* (AY065887.1), *Alouatta macconnelli* (AY065888.1), *Alouatta guariba guariba* (AY065899.1), *Alouatta guariba clamitans* (DQ679782.1), *Alouatta pigra* (AY065884.1), *Alouatta palliata* (AY065879.1) (Cortés-Ortiz et al. 2003, Harris et al. 2005, Lorenz et al. 2005, Nascimento et al. 2005, Casado et al. 2010). *Cebus apella* Linnaeus, 1758 (FJ529102.1) and *Lagothrix lagotricha* Humboldt, 1812 (AY671799.1) were used as outgroups. *Cebus apella*, from the Cebidae family, was taken as an outgroup species, since it is accepted that this species presents the most ancestral karyotype within Platyrrhini (Clemente et al. 1990, García et al. 2000). *Lagothrix lagotricha*, also a member of the Atelidae family, was chosen as the second outgroup to test the monophyly of the group. All sequences were aligned using CLUSTALW (Thompson et al. 1994).

*Phylogeny*: A Maximum Parsimony phylogeny using the exhaustive search option was obtained with PAUP 4.0 software (Phylogenetic Analysis Using Maximum Parsimony, (Swofford 2002)), for each separate partition and the combination of both the chromosomal and molecular datasets. All characters had the same weight, based on the premise that chromosome rearrangements occur by equal chance (de Oliveira et al. 2002, Dobigny et al. 2004). The relative stability of nodes was assessed by bootstrap estimates (Felsenstein 1985) based on 200 iterations. Each bootstrap replicate involved a heuristic parsimony search with 10 random taxon additions and tree-bisection reconnection (TBR) branch swapping.

## Results

### Classical cytogenetic analysis

**Figure 1.**
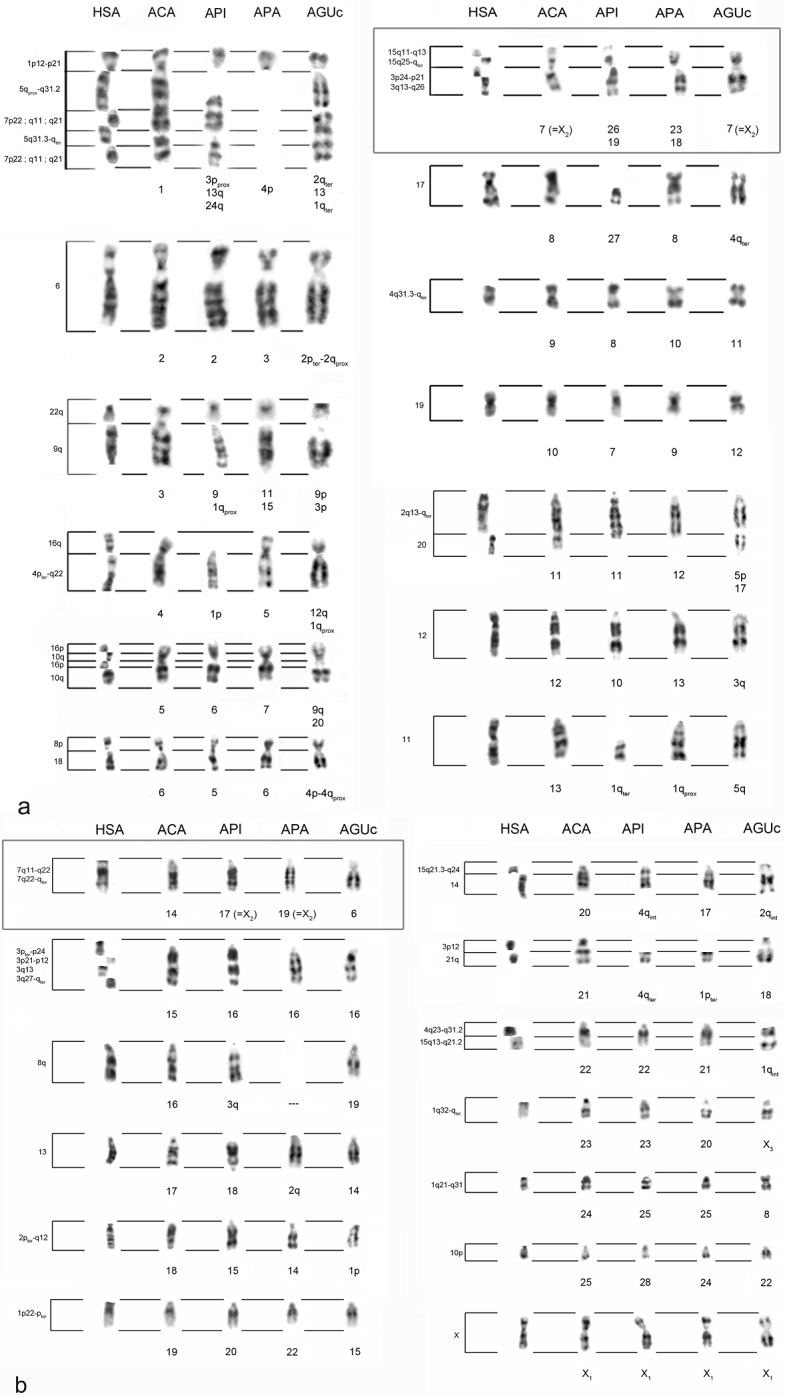
Comparison of *Homo sapiens* (HSA), *Alouatta caraya* (ACA), *Alouatta pigra* (API), *Alouatta palliata* (APA) and *Alouatta guariba clamitans* (AGUc) G-banded chromosomes, taking *Alouatta caraya*’s karyotype as reference. On the left, human chromosomal bands with homeology for its corresponding ACA chromosome segment are indicated. The boxes highlight the homeologies of the autosomes involved in the sex chromosome systems in these species **a** Comparison for ACA chromosomes #1 to #13 **b** Comparison for ACA chromosomes #14 to X_1_

*Karyological analysis*: The cytogenetic characterization of the *Alouatta* specimens showed diploid numbers, sex chromosome systems and G-bandings patterns in agreement with the ones previously described for each species. Figures 1a and 1b show all the comparisons performed.

*Chromosomal homeologies between Alouatta caraya and Alouatta palliata*: The chromosomal rearrangements that could explain the homeologies were grouped in two categories: 1) *Alouatta palliata* chromosomes with no rearrangements with respect to *Alouatta caraya* chromosomes: 3, 5, 6, 7, 8, 9, 10, 13, 14, 16, 17, 19, 20, 21, 22, 24, 25 and X_1_; 2) *Alouatta palliata* chromosomes with more than one rearrangement with respect to *Alouatta caraya* chromosomes: 1, 2, 4, 11, 12, 15, 18 and 23. No homeologies were allocated for *Alouatta palliata* chromosome 26 and chromosome arms 4q and 2p using the level of resolution of the classical cytogenetic techniques applied. The rearrangements detected between the *Alouatta caraya* and *Alouatta palliata* karyotypes included at least seven fissions/fusions, two paracentric inversions and one deletion. *Alouatta caraya* chromosome 7 (X_2_ in males) shares homeology with two *Alouatta palliata* chromosome pairs, 23 and 18, which are not the ones involved in the sex chromosome system in *Alouatta palliata*. The *Alouatta palliata* chromosomal pair 19 (X_2_ in males) shares homeology with chromosome 14 of *Alouatta caraya*.

*Chromosomal homeologies between Alouatta caraya and Alouatta pigra*: The chromosomal rearrangements that could explain the homeologies were grouped in two categories: 1) *Alouatta pigra* chromosomes with no rearrangements with respect to *Alouatta caraya* chromosomes: 2, 5, 6, 7, 8, 10, 15, 16, 17, 20, 22, 23, 25, 28 and X_1_; 2) *Alouatta pigra* chromosomes with more than one rearrangement with respect to *Alouatta caraya* chromosomes: 1, 3, 4, 9, 11, 13, 18, 19, 24, 26 and 27. No homeologies were allocated for *Alouatta pigra* chromosomes 4p_prox_, 12, 14 and 21 using the level of resolution of the classical cytogenetic techniques applied. The rearrangements detected between the *Alouatta caraya* and *Alouatta pigra* karyotypes included at least 12 fissions/fusions, two paracentric inversions, two translocations and one deletion. *Alouatta caraya* chromosome 7 (X_2_ in males) shares homeology with two *Alouatta pigra* chromosome pairs, 26 and 19, which are not the ones involved in the sex chromosome system in *Alouatta pigra*. *Alouatta pigra* chromosome 17 (X_2_ in males) shares homeology with chromosome 14 of *Alouatta caraya* (which in turn has homeology with HSA7).

*Chromosomal homeologies among all howlers*: The chromosomal homeologies found among all howlers are shown in Table 3 and Figures 1a and 1b. Results show that Mesoamerican howlers share several human chromosomal syntenic associations with South American ones: HSA15q13-q21.2/4q23-q31.2 and HSA16p/10q, shared with all howlers; HSA15q21.3-q24/14, shared with all howlers except *Alouatta seniculus arctoidea* and *Alouatta macconnelli*, and HSA8p/18, shared with all howlers except *Alouatta seniculus arctoidea*. Two new chromosomal syntenic associations, HSA4p_ter_-q22/9/11 and HSA15q21.3-q24/14/21q, were found for *Alouatta pigra* in chromosomes 1 and 4q, respectively.

**Table 3.** Chromosomal homeologies between howlers, obtained from data both from this contribution and from previous reports. ACA: *Alouatta caraya*; API: *Alouatta pigra*; APA: *Alouatta palliata*; AGU: *Alouatta guariba*; ASEa: *Alouatta seniculus arctoidea*; AMA: *Alouatta macconnelli*; ASA: *Alouatta sara*; ABE: *Alouatta belzebul*.

Human Chromosomal associations†	ACA	API	APA	AGU	ASEa	AMA	ASA	ABE
1p12-p21	1	3p_prox_	4p	2q_ter_	9q_ter_	18q_ter_	16q_ter_	23
5q_prox_-q31.2				13	3q_ter_	13	1q_ter_	1q
7p22; q11; q21		13q		1q_ter_	12q_prox_	12q_ter_	13q_ter_	
5q31.3-q_ter_					8q_ter_		7q_ter_	
7p22; q11; q21		24q			1q_ter_		4q_ter_	
6	2	2	3	2p_ter_-q_prox_	4	18p_ter_-q_prox_	5	4
						8		
22q	3	9	11	9p	9q_prox_	5p_prox_	8q_prox_	6p_ter_
9q		1q_prox_	15	3p	13	15	11	2q
16q	4	1p	5	12q	6q_ter_	3q	9p_ter_	5
4p_ter_-q22				1q_prox_	11		14	
16p	5	6	7	9q	10	3p_prox_	21	7
10q				20		2p	19	
16p								
10q								
8p	6	5	6	4p_ter_-q_prox_	15q_ter_	6	2q_ter_	8
18					5q_prox_			
15q11-q13	7 (X_2_)	26q	23	7 (X_2_)	X_2_	X_2_	X_2_	24
15q25-q_ter_								
3p24-p21		19q	18					17 (X_2_)
3q13-q26								
17	8	27q	8	4q_ter_	7q_prox_	7	1p-1q_prox_	9
					2p			
4q31.3-q_ter_	9	8	10	11	18	10	16p-q_prox_	11
19	10	7	9	12	7q_ter_	5p_ter_	15p-q_prox_	10
						4p		
2q13-q_ter_	11	11	12	5p	2p_prox_	11q_prox_	3q_prox_	12
20				17	9q_int_	16q_prox_	8q_int_	
12	12	10	13	3q	2	14	6	2p
11	13	1q_ter_	1q_prox_	5q	3q_prox_	2q	1q_int_	1p
					12q_ter_		13q_prox_	
7 q11-q21	14	17 (X_2_)	19 (X_2_)	6	8	1q	7	13
7q22-q_ter_								
3p_ter_-p24	15	16	16	16	6q_prox_	19	2q_prox_	15
3p21-p12								
3q13								
3q27-q_ter_								
8q	16	3q	--	19	1q_prox_	12q_prox_	4q_prox_	16
13	17	18	2q	14	16	4q	12	14
2p_ter_- q12	18	15	14	1p	2q_prox_	17	10	3q
1p21-p_ter_	19	20	22	15	14	5q	17	3p
15q21.3-q24	20	4q_int_	17	2q_int_	6q_int_	1p	9q_prox_	6p_prox_-q_ter_
14					5q_ter_		18	
3p12	21	4p_ter_	1p_ter_	18	17	9	20	18q_ter_
21q								
4q23-q31.2	22	22	21	1q_int_	1p_ter_	1q_ter_	3q_ter_	20
15q13-q21.2								
1q32-q_ter_	23	23	20	X_3_	15q_prox_	21	8q_ter_	19
1q21-q31	24	25	25	8	3p	16q_ter_	9q_ter_	21
10p	25	28	24	22	7q_int_	3p_ter_	15q_ter_	22
X	X_1_	X_1_	X_1_	X_1_	X_1_	X_1_	X_1_	X_1_

† from pter to qter.

### Cytomolecular study

**Figure 2.**
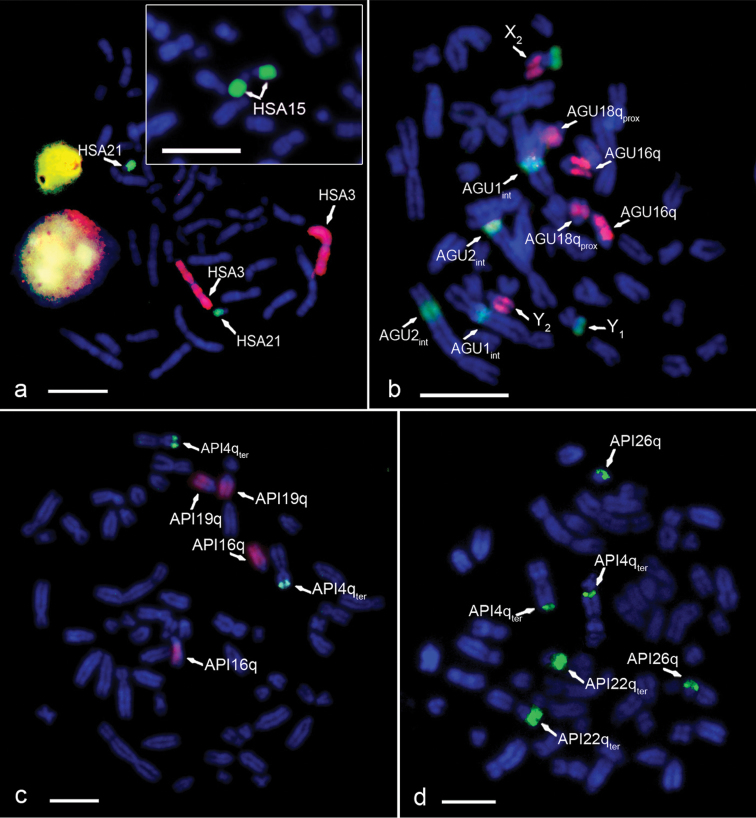
Analysis of the conservation of the HSA3/21 and HSA3/15 syntenic chromosomal associations in howlers (bar=10 μm). The arrows indicate the chromosomes with positive FISH signal **a** *Homo sapiens* partial metaphase hybridized with probes HSA21 (green) and HSA3 (red) (control of the hybridization). Inset: *Homo sapiens* partial metaphase hybridized with HSA15 (green) **b** AGUc metaphase hybridized with HSA15 (green) and HSA3 (red) **c** API metaphase hybridized with HSA3 (red) and HSA21 (green) **d** API metaphase hybridized with HSA15 (green).

In the *Homo sapiens* metaphases, the hybridization signals on chromosomes HSA3, HSA21, HSA15, HSAX and HSAY for chromosome painting probes #3 (red), #21 (green), #15 (green) (Figure 2a), X and Y (data not shown) were corroborated.

**Figure 3.**
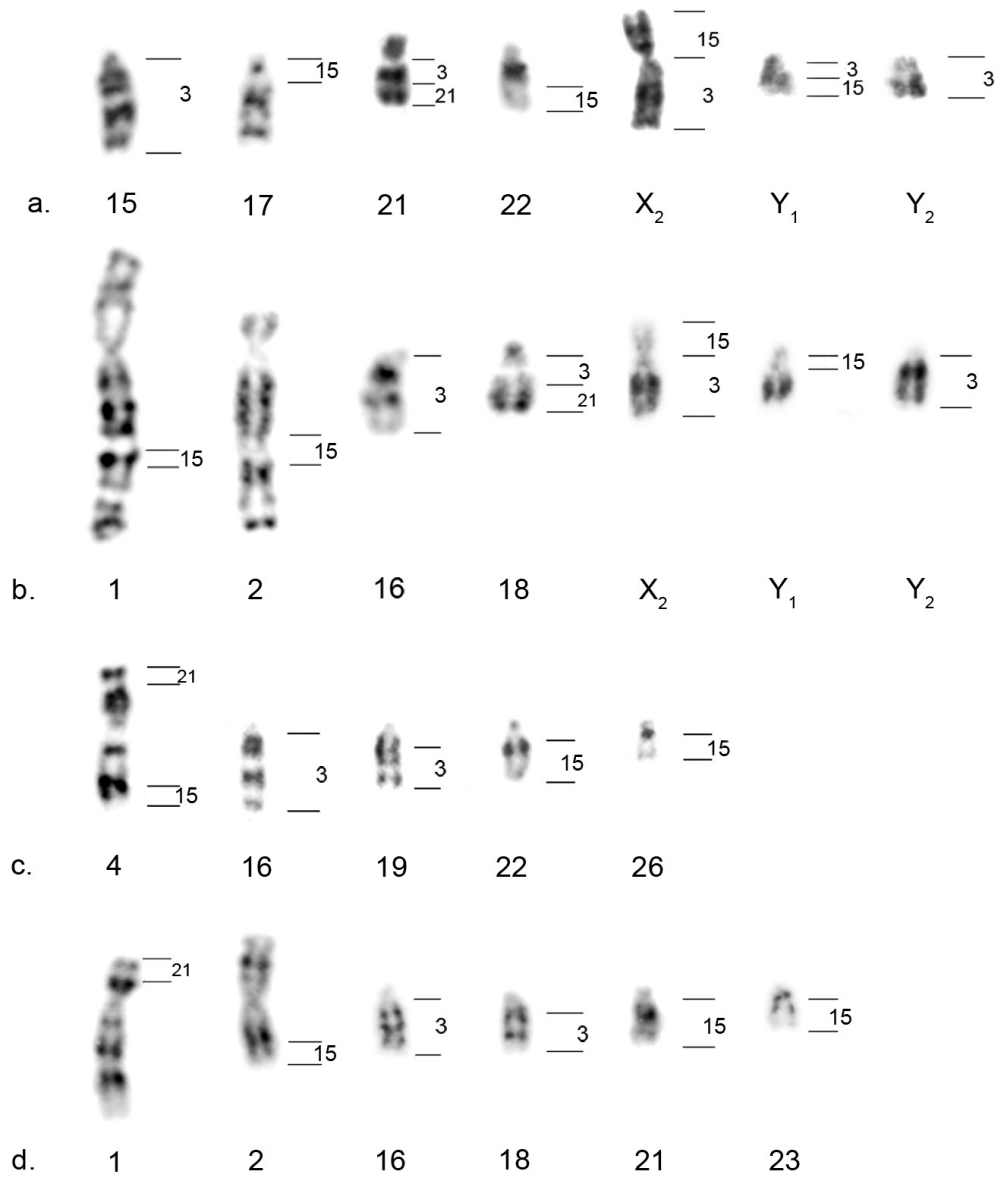
Howler monkeys G-banded chromosomes with positive signal for the human chromosome painting probes analyzed. On the right, the hybridization pattern of human chromosomes #3, #21 and #15. **a** *Alouatta caraya* **b** *Alouatta guariba clamitans* **c** *Alouatta pigra* **d** *Alouatta palliata*.

In *Alouatta guariba clamitans*, the signal for HSA21 was observed in 18q_ter_, the signal for HSA3 was observed in 18q_prox_ (thus corroborating the HSA3/21 synteny in *Alouatta guariba clamitans*), 16q, 7q (X_2_ in males) and Y_2_, and the signal for HSA15 was observed in 1_int_, 2_int_, 7p (X_2_) and Y_1_. This corroborates the HSA3/15 syntenic association to the multiple sex chromosome system X_1_X_1_X_2_X_2_X_3_X_3_/X_1_X_2_X_3_Y_1_Y_2_ of this species (Figures 2b and 3b).

In *Alouatta pigra*, the signal for HSA3 was observed in 16q and 19q, while that for HSA21 hybridized in 4p_ter_, thus indicating that the HSA3/21 synteny is not present in *Alouatta pigra* (Figures 2c and 3c). The probe for HSA15 hybridized in *Alouatta pigra* metaphases in 4q_ter_, 22q_ter_ and 26q, showing that the HSA3/15 syntenic association is also absent. None of these *Alouatta pigra* chromosomes is involved in the sex chromosome system of this species (Figures 2d and 3c).

In *Alouatta caraya*, the signal for HSA21 was observed in 21q_ter_, whereas that for HSA3 was observed in 21q_prox_, thus confirming the conservation of the HSA3/21 synteny. HSA15 hybridized in 7p (X_2_ in males) and Y_1ter_, and HSA3 in 7q and Y_1prox_, exhibiting the HSA3/15 syntenic association in the sex chromosome system X_1_X_1_X_2_X_2_/X_1_X_2_Y_1_Y_2_ (Figure 3a).

*Alouatta palliata* showed a pattern similar to that of *Alouatta pigra* (therefore Figure 2 illustrates only the latter). HSA3 hybridized in 16q and 18q, HSA21 hybridized in 1p_ter_ and HSA15 in 2q_ter_, 21q_ter_ and 23q (Figure 3d). Both the HSA3/21 and HSA3/15 syntenic associations are absent in *Alouatta palliata* and chromosomes with homeology to HSA3 and HSA15 are also not involved in the sex chromosome system of this species.

The probe for the human X chromosome showed positive hybridization signal in X_1_ of all the species analyzed. The probe for the human Y chromosome did not hybridize in any of the howler species (data not shown).

### Phylogenetic analysis

The data obtained from the G-banding pattern and FISH homeologies, together with cyt b sequences obtained from previous reports, were used as the basis to perform a cladistic analysis. The HSAY/7 association, corresponding to the Y-autosome translocation that gave rise to the multivalents observed in *Alouatta pigra* and *Alouatta palliata*, was added as an extra character to the original list (de Oliveira et al. 2002). The syntenic associations HSA4p_ter_-q22/9/11 observed in chromosome 1 of *Alouatta pigra* and HSA15q21.3-q24/14/21q observed in chromosome arm 4q were not included in the analysis, because, as autopomorphies for *Alouatta pigra*, they are considered non-informative.

Three data matrices were obtained: one including only chromosomal data, another including only molecular data and the last one including both types of characters (chromosomal and molecular) in a single matrix (see Appendix 1).

*Chromosomal partition*: The analysis of chromosomal data resulted in 36 informative characters, 23 constant characters and 40 non-informative characters. After analyzing 704 trees, PAUP retained the two most parsimonious trees (Appendix 2: Figures Sa and Sb), both with a length of 87 (L = 87). The analysis using only the partition of chromosomal data did not resolve the node ((*Alouatta palliata*, *Alouatta pigra*), (*Alouatta caraya*, *Alouatta belzebul*), ((*Alouatta guariba clamitans*, *Alouatta guariba guariba*), (*Alouatta macconnelli* (*Alouatta sara*, *Alouatta seniculus arctoidea*))), since it was established in a polytomy (Appendix 2: Figure Sc).

*Molecular partition*: Heuristic analysis of cyt b gene sequences, made ​​from a total of 800 characters, produced 109 informative characters, 551 constant characters and 140 non-informative characters. After analyzing 916 trees, PAUP retained a single most parsimonious tree (Appendix 2: Figure Sd), with a length of L = 366. The analysis using only molecular data did not resolve the node (*Alouatta sara*, *Alouatta macconnelli*, *Alouatta seniculus arctoidea*, *Alouatta caraya*), which was established as a polytomy different from that described from chromosomal data.

**Figure 4.**
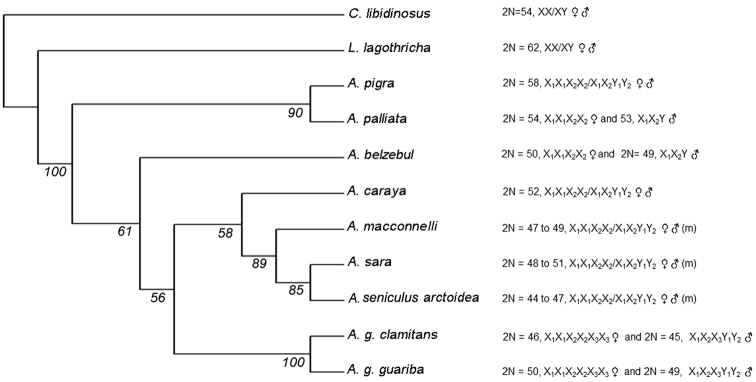
50% majority consensus tree obtained by “bootstrap” for the combined analysis. Next to the name of each species, the diploid number (2N) and sex chromosome system is described. (m)=microchromosomes.

*Combined analysis*: The heuristic analysis of the combined data showed a total of 899 characters, 145 of which were informative, 180 non-informative and 574 constant. After analyzing 684 trees, PAUP retained only one, with a length of L=460 (Figure 4). This type of analysis allowed us to solve all the nodes, resulting in a fully resolved tree.

## Discussion

We present the first phylogenetic study using a combined analysis of chromosomal and molecular characters in Ceboidea to contribute to the characterization of the speciogenic processes in howler monkeys. The homoplasy distribution is likely to be different in each dataset because these are subject to different constraints. Therefore, when different datasets are analyzed simultaneously, the signal common to all of them is more likely to overwhelm the homoplasy signal on the data (Kluge 1989).

In primates, few studies have compared and taken into account more than one type of character. Bonvicino et al. (2001) superimposed chromosomal information on the phylogeny obtained from molecular characters. Villalobos et al. (2004) used numerical and metric values that describe the karyotype, such as diploid number (2N) and fundamental number (FN), in a combined phylogenetic analysis with morphological characters. However, these values (2N, FN, etc) can be identical simply by chance and, if interpreted in a phylogenetic context, may be spurious indicators of relatedness (Dobigny et al. 2004). Our encoding strategy (using the rearrangements as characters) is quite similar to that used for morphological data but in cytogenetics one can retrieve information on the mutational event itself, something that is clearly not available to morphologists. As such, chromosomal mutations that accumulate along the tree are comparable to transitions, transversions, and insertions/deletions in molecular phylogenies (Dobigny et al. 2004). Our combined phylogeny evidences the accuracy of this encoding strategy.

In all the above-mentioned contributions, the X_1_X_1_X_2_X_2_/X_1_X_2_Y_1_Y_2_ sex chromosome system was proposed as the ancestral condition for the genus. However, as discussed by Solari and Rahn (2005), the X_1_X_1_X_2_X_2_/X_1_X_2_Y sex chromosome system is simpler and is present in other genera of Neotropical Primates, such as *Aotus* Illiger, 1811, *Callimico* Miranda Ribeiro, 1912, and *Cacajao* Lesson, 1840 (Ma et al. 1976, Seuánez et al. 1989, Moura-Pensin et al. 2001). The X_1_X_1_X_2_X_2_/X_1_X_2_Y sex chromosome system as an ancestral state appears to be a more parsimonious hypothesis. Moreover, since Mesoamerican howlers (*Alouatta pigra* and *Alouatta palliata*) were poorly karyologically characterized at the time, data on these howlers are missing in all previous contributions.

### Homeology analysis

The karyotypes of *Alouatta pigra* and *Alouatta palliata* share more syntenic associations with those of *Alouatta caraya* and *Alouatta belzebul* than with those of the “*Alouatta seniculus* group” (*Alouatta seniculus arctoidea*, *Alouatta sara*, *Alouatta macconnelli*, denominated as such because they were once all subspecies of *Alouatta seniculus* together with *Alouatta seniculus seniculus* Linnaeus, 1766, and *Alouatta seniculus stramineus* Hill, 1962). This supports the basal grouping of the *Alouatta pigra*-*Alouatta palliata* Mesoamerican clade and the basal grouping of *Alouatta belzebul* among South American howlers.

The chromosomal comparisons showed that *Alouatta pigra* and *Alouatta palliata* conserved the HSA8/18 and HSA14/15 syntenies, considered ancestral for Platyrrhini (Stanyon et al. 2008), as well as the HSA10/16/10/16 syntenic association, ancestral for Atelidae (de Oliveira et al. 2002), but lost the HSA3/21 synteny, ancestral for mammals (Müller et al. 2000, Müller 2006).

According to our combined phylogeny, the HSA2/20 and HSA5/7/5/7 syntenic associations, previously considered as synapomorphies of the *Alouatta caraya*-*Alouatta belzebul* group (de Oliveira et al. 2002), would be homoplasies (parallelism). The HSA16/4 syntenyc association would be ancestral for the genus and might either be absent in *Alouatta pigra*, *Alouatta guariba* and the *Alouatta seniculus* group or might constitute a parallelism among *Alouatta palliata*, *Alouatta caraya* and *Alouatta belzebul*.

Like the HSA3/21 synteny, the HSA3/15 syntenic association, involved in the sex chromosome systems in South American howlers, is not present in Mesoamerican ones. This syntenic association of human 3/15 chromosomal segments has been described in other Atelidae species such as *Ateles geoffroyi* Kuhl, 1820 and *Ateles belzebul hibridus* Geoffroy, 1829, although not associated with the sex chromosome system (Morescalchi et al. 1997), but not observed in *Lagothrix* Geoffroy, 1812, and *Brachyteles* Spix, 1823 (Stanyon et al. 2001, de Oliveira et al. 2005). This association has not been observed in other genera of Neotropical Primates such as *Cebus libidinosus* Spix, 1823, or *Saimiri boliviensis boliviensis* Geoffroy & Blainville, 1834 (Mudry et al. 2001). Therefore, the HSA3/15 syntenic association either could be interpreted as the ancestral condition for the family Atelidae, where the association with multiple sex chromosomes would be an evolutionary novelty (apomorphy) in howlers and the loss of the association a apomorphy for the *Lagothrix* and *Brachyteles* group, or could have appeared independently in *Alouatta*, involved in the Y-autosome translocation, and in *Ateles*, not involved in the sex chromosome system (de Oliveira et al. 2005). However, our results suggest that the HSA3/15 syntenic association is not an ancestral condition for *Alouatta*, since the most basal species (see Figure 4) *Alouatta pigra* and*Alouatta palliata* (this contribution) and *Alouatta belzebul* (Consigliere et al. 1996) do not possess this association.

### Possible origin of the multivalents

Taking into consideration the data obtained, a hypothesis can be proposed regarding the origin of the sex chromosome systems in the genus. Within the family Atelidae, with the exception of *Alouatta*, all genera have an XX/XY sex chromosome system. Therefore, it can be considered that the *Alouatta* ancestor possessed a chromosomal sex determination XX/XY, prior to the biogeographic separation of Mesoamerican and South American groups (see below). After this separation, both groups independently acquired the multiple sex chromosome systems currently observed through independent Y-autosome translocations.

**Figure 5.**
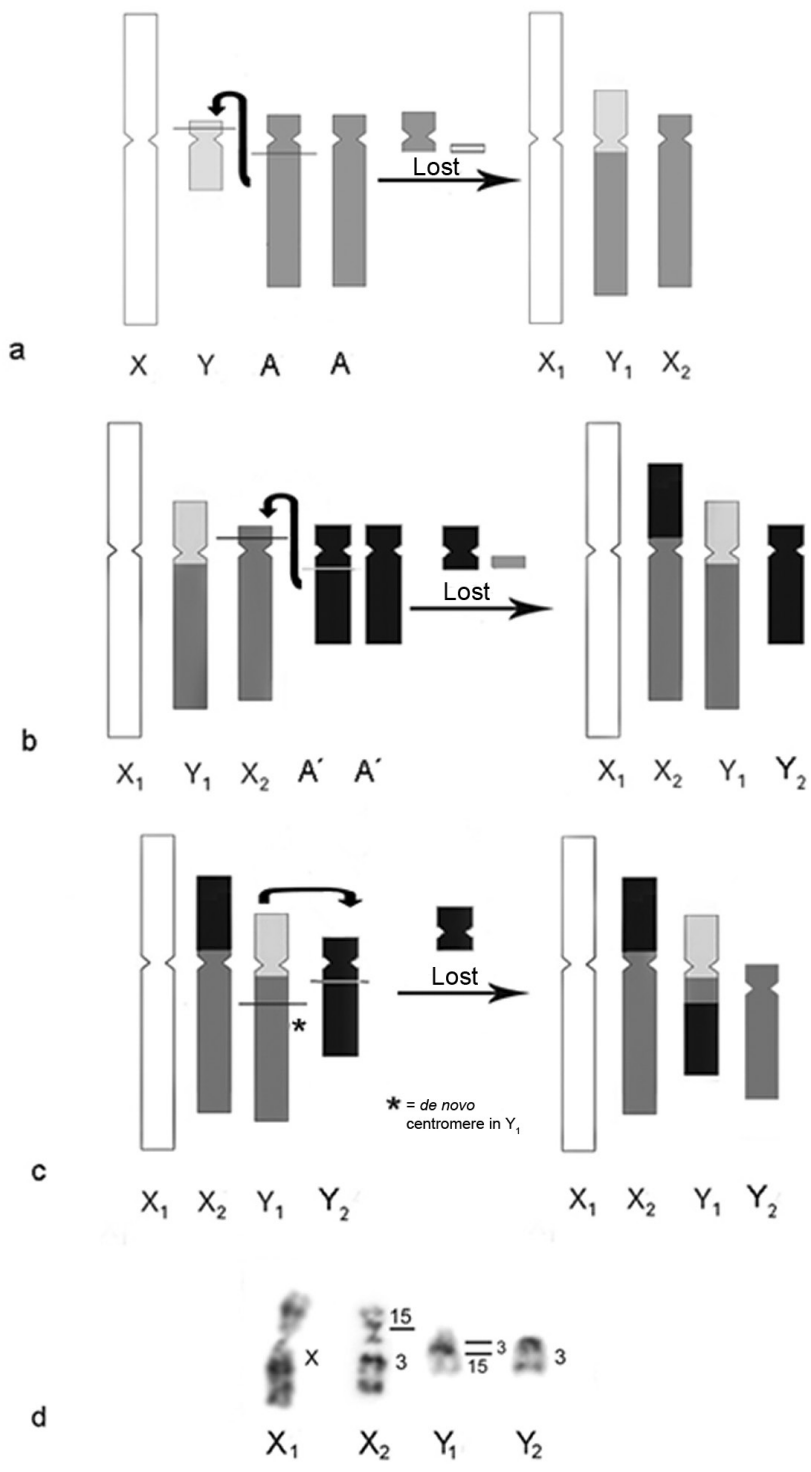
**a** Possible origin for X_1_X_1_X_2_X_2_/X_1_X_2_Y sex chromosome system in the genus *Alouatta*. The ancestral X chromosome is shown in white, the ancestral Y chromosome in light gray and the autosomal pair (A) in dark gray. Two fissions occurs, one in Yp_ter_ and another in q_prox_ of the autosomal pair involved (Aq_prox_). The translocation of Yq-p_prox_ to the Aq formed the new Y_1_ chromosome and the homolog of the autosomal pair involved in the translocation is now denominated X_2_. The Yp_ter_ acentric fragment is lost and the rest of the autosome (Ap and Aq_prox_) could either be lost or remain as a microchromosome in some howlers **b** Possible origin for the X_1_X_1_X_2_X_2_/X_1_X_2_Y_1_Y_2_ sex chromosome systems from a X_1_X_1_X_2_X_2_/X_1_X_2_Y system. The ancestral X is shown in white, the ancestral Y in light gray, the autosomal pair involved in the first translocation (A) in dark gray and the autosomal pair (A´) involved in the formation of this new sex chromosome system in black. Simultaneous breaks in X_2_p_prox_ and A`q_prox_ followed by the translocation of the rest of the A`q to X_2_p_prox_ give origin to the new X_2_ chromosome. The X_2_p_ter_ acentric fragment could be lost and the rest of the autosome (A´) could either be lost or remain as a microchromosome in some howlers. The homolog to the autosomal chromosome in question is now identified as Y_2_ **c** Simultaneous breaks in Y_1_q and Y_2_q and a translocation between Y_1_ and Y_2_ further explain the hybridization pattern observed in the sex chromosome systems of South American howlers. A *de novo* centromere arises in the remains of the old Y_1_ (now Y_2_). The remains of the old Y_2_ could either be lost or remain as a microchromosome in some howlers **d** Hybridization pattern in South American howlers.

The sex chromosome system X_1_X_1_X_2_X_2_/X_1_X_2_Y would have arisen independently in the lineages of Meso and South American howlers by a Y-autosome translocation (Figure 5a). In males, two fissions, one in Yp_ter_ and another in q_prox_ of the autosomal pair involved (Aq_prox_), followed by translocation of Aq_prox_ to Yq-p_prox_, formed the new chromosome Y_1_. The Yp_ter_ segment is lost and the proximal region of the fissioned autosome either is lost or, in certain howler species, could have given rise to microchromosomes (e.g.: *Alouatta seniculus* (Yunis et al. 1976, Lima and Seuánez 1991, Torres and Leibovici 2001), *Alouatta sara* (Minezawa et al. 1985) and *Alouatta macconnelli* (Lima et al. 1990)). The homologous autosomal pair involved in the translocation is the one now denominated X_2_. In the case of South American howlers, the autosomal pair involved would share homeology with HSA3, whereas in the Mesoamerican species it would share homeology with HSA7.

From this X_1_X_1_X_2_X_2_/X_1_X_2_Y sex chromosome system, an X_1_X_1_X_2_X_2_/X_1_X_2_Y_1_Y_2_ system could have arisen from a new translocation (Figure 5b). Under this hypothesis, simultaneous breaks in X_2_p_prox_ and q_prox_ of another autosome (A´q_prox_), followed by the translocation of most of the A´q arm to X_2_p_prox_, gave rise to the new X_2_ chromosome. The X_2_p_prox_ acentric fragment is lost and the rest of the autosome (A´) either is lost or could have remained as a microchromosome in some howler species (see above). The chromosome homologous to the autosome in question (A`) became Y_2_. In the case of South American howlers, the new autosomal pair involved in the sex chromosome system would share homeology with HSA15. A further translocation between Y_1_ and Y_2_ (Figure 5c) would explain the hybridization pattern of the segments with homeology to human chromosomes 3 and 15 observed in the sex chromosome systems X_1_X_1_X_2_X_2_/X_1_X_2_Y_1_Y_2_ in South American howlers (Figure 5d).

On the other hand, in the Mesoamerican species, the X_1_X_1_X_2_X_2_/X_1_X_2_Y_1_Y_2_ sex chromosome system could have arisen either as described in Figure 5b (with the autosomal pair involved sharing homeology with a human chromosome not yet identified by G-banding pattern) or by a fission in Y_1_ that would have given rise to two chromosomes, the new Y_1_ (containing the segment corresponding to the ancestral Y chromosome) and Y_2_ (containing a portion of the autosomal pair with homeology to HSA7). This last hypothesis would require a centromeric activation in Y_2_.

However, considering the observation of the independent origin of the multiple sex chromosome systems in these two groups of howlers, the possibility of an independent origin of the X_1_X_1_X_2_X_2_/X_1_X_2_Y and X_1_X_1_X_2_X_2_/X_1_X_2_Y_1_Y_2_ sex chromosome systems within the Meso and South American groups cannot be ruled out until further studies.

It can be considered that multiple sex chromosome systems would be an extremely rare phenomenon due to complication in meiosis. Extreme cases are platypus and echidna, with a large number of sex chromosomes (Bick and Jackson 1967, Renz et al. 2007). In primates, multiple sex chromosome systems are even more infrequent. Moreover, *Alouatta* would be the first case where an independent origin of multiple sex chromosome systems is described. In other taxa, such as *Drosophila* (Flores et al. 2008), Erythrinidae fishes (Cioffi et al. 2013) and mole-rats (Deuve et al. 2006), a few cases have been observed, but these descriptions are still scarce.

### Phylogeny of *Alouatta*

The chromosomal homeologies and FISH analysis were used to construct a data matrix for the phylogenetic analysis. For comparison purposes, independent phylogenetic reconstructions were performed with each type of partition (Appendix 2: Figure Sa, b, c and d), along with the combined analysis of the two datasets (Figure 4). The chromosome partition grouped *Alouatta caraya* and *Alouatta belzebul* as sister taxa, in agreement with that reported by de Oliveira et al. (2002), a relationship that was not observed in the other two analyses, which grouped *Alouatta caraya* with *Alouatta sara*, *Alouatta seniculus arctoidea* and *Alouatta macconnelli* (although in the case of the molecular partition this relationship constituted a polytomy). This last species arrangement was also proposed by Nascimento et al. (2005) and Cortés-Ortiz et al. (2003) using molecular characters. In our molecular data partition (Figure Sd), *Alouatta belzebul* was grouped with the clade of *Alouatta guariba*, in agreement with that reported by Bonvicino et al. (2001) and Cortés-Ortiz et al. (2003). The three types of analyses agreed to place *Alouatta sara*, *Alouatta seniculus arctoidea* and *Alouatta macconnelli* into a single group, although the molecular data partition did not resolve the relationships between them, as they formed a polytomy. The grouping of all those species (“*Alouatta seniculus* group”) was observed in all phylogenetic studies performed so far (see above). Another coincidence was that the Mesoamerican species were placed as a separate clade from other South American species and, as expected, the two subspecies of *Alouatta guariba* in one group. All sets of taxa analyzed in this new approach were solved without polytomies only with the combined analysis, demonstrating the usefulness of incorporating more than one source of data for a more accurate elucidation of the relationships among current taxa.

The grouping of South American species as a separate group of the Mesoamerican group coincides with previous phylogenetic analyses using only molecular characters (Cortés-Ortiz et al. 2003, Ellsworth and Hoelzer 2006) and with the hypothesis of monophyletic origin of the Mesoamerican howlers previously proposed by Smith (1970). Smith’s hypothesis holds that Mesoamerican howlers originated by an expansion of the geographic distribution of South American howlers after the formation of the Isthmus of Panama, estimated to be completed about 3 million years ago (Coates et al. 2004). However, other studies indicate that the rise of the isthmus was a process rather than an event (Knowlton and Weigt 1998), resulting in intermittent periods with connected and divided lands during the past 18 million years. Given this last fact, another hypothesis was postulated to explain the current geographic distribution of the species of the genus. Instead of a single colonization event, various founder events, either across the Isthmus of Panama during one of the periods in which the two Americas were connected or across islands in the Caribbean archipielago, could have occurred (Cortés-Ortiz et al. 2003, Ellsworth and Hoelzer 2006). Primate fossils have been found in Cuba and Jamaica, but the origin and relationships of these specimens with modern Platyrrhini are still under debate (Fleagle 1999, Gutiérrez Calvache and Jaimez Salgado 2007, Rosenberger et al. 2009, Cooke et al. 2011).

Independently of the biogeographic scenario under consideration, it is clear that the evolutionary history of Mesoamerican howlers is different from that of South American howlers, an assertion that would be supported by the evidence provided by our new data.

This contribution provides new useful information for the systematics of the genus *Alouatta*, while supporting the hypothesis of chromosomal evolution in primates as a speciogenic strategy. The combined analysis resolved the phylogenetic relationships between howler species of both American origins, as a first approach to the “Total Evidence” concept and towards clarifying the controversies related to the Taxonomy and Evolution of Ceboidea.

## Appendix 1

Data matrix. (doi: 10.3897/CompCytogen.v8i1.6716.app1) File format: Microsoft Word file (doc). **Explanation note:** Data matrix contains: Chromosomal data matrix, Molecular data matrix and Combined data matrix.

## Appendix 2

Supplementary Figure S. (doi: 10.3897/CompCytogen.v8i1.6716.app2) File format: Microsoft Word file (doc). **Explanation note:** Figure S: a) and b) Most parsimonious trees obtained for the chromosomal partition c) 50% majority consensus tree obtained by “bootstrap” d) 50% majority consensus tree obtained by “bootstrap” for the molecular partition.
