# Supplementary material for: Multiple sex chromosome systems in howler monkeys (Platyrrhini, Alouatta)
Source: Comp Cytogenet. 2014 Feb 25;8(1):43–69. doi: 10.3897/CompCytogen.v8i1.6716 (PMC3978242; doi:10.3897/CompCytogen.v8i1.6716)
Supplement: Supplementary file 1 — Data matrix. (doi: 10.3897/CompCytogen.v8i1.6716.app1) File format: Microsoft Word file (doc). [file CompCytogen-008-043-s001.doc]

**Data matrix obtained for each partition**

-Chromosomal data matrix

#Nexus

Begin data;

dimensions ntax=11 nchar=99;

matrix

| *A. guariba guariba* | 0 | 0 | 0 | 0 | 0 | 1 | 0 | 1 | 1 | 0 | 1 | 1 | 1 | 1 | 1 | 1 | 1 | 1 | 1 | 1 | 1 | 1 | 1 | 1 | 1 | 1 | 1 | 1 | 1 | 1 | 1 | 1 | 1 | 1 | 0 | 1 | 1 | 1 | 1 | 0 |
| --- | --- | --- | --- | --- | --- | --- | --- | --- | --- | --- | --- | --- | --- | --- | --- | --- | --- | --- | --- | --- | --- | --- | --- | --- | --- | --- | --- | --- | --- | --- | --- | --- | --- | --- | --- | --- | --- | --- | --- | --- |
| *A. guariba clamitans* | 0 | 0 | 0 | 0 | 0 | 1 | 0 | 1 | 1 | 0 | 1 | 1 | 1 | 1 | 1 | 1 | 1 | 1 | 1 | 1 | 1 | 1 | 1 | 1 | 1 | 1 | 1 | 1 | 1 | 1 | 1 | 1 | 1 | 1 | 0 | 1 | 1 | 1 | 1 | 0 |
| *A. sara* | 0 | 0 | 0 | 0 | 0 | 0 | 0 | 1 | 1 | 0 | 1 | 1 | 1 | 1 | 1 | 1 | 1 | 1 | 1 | 1 | 1 | 1 | 1 | 1 | 1 | 1 | 1 | 1 | 1 | 1 | 1 | 1 | 1 | 0 | 0 | 1 | 1 | 1 | 1 | 0 |
| *A. macconnelli J* | 0 | 0 | 0 | 0 | 0 | 0 | 0 | 1 | 1 | 0 | 1 | 1 | 1 | 1 | 1 | 1 | 1 | 1 | 1 | 1 | 1 | 1 | 1 | 1 | 1 | 1 | 1 | 1 | 1 | 1 | 1 | 1 | 1 | 1 | 0 | 1 | 1 | 1 | 1 | 0 |
| *A. s.arctoidea* | 0 | 0 | 0 | 0 | 0 | 0 | 0 | 1 | 0 | 0 | 1 | 1 | 1 | 1 | 1 | 1 | 1 | 1 | 1 | 1 | 1 | 1 | 1 | 1 | 1 | 1 | 1 | 1 | 1 | 1 | 1 | 1 | 1 | 0 | 1 | 1 | 1 | 1 | 1 | 0 |
| *A. caraya* | 0 | 0 | 0 | 0 | 0 | 0 | 0 | 1 | 1 | 0 | 1 | 1 | 1 | 1 | 1 | 1 | 1 | 1 | 1 | 1 | 1 | 1 | 1 | 1 | 1 | 1 | 1 | 1 | 1 | 1 | 1 | 1 | 1 | 1 | 1 | 1 | 1 | 1 | 1 | 0 |
| *Alouatta belzebul* | 0 | 0 | 0 | 0 | 0 | 0 | 0 | 1 | 1 | 0 | 1 | 1 | 1 | 1 | 1 | 1 | 1 | 1 | 1 | 1 | 1 | 1 | 1 | 1 | 1 | 1 | 1 | 1 | 1 | 1 | 1 | 1 | 1 | 1 | 1 | ? | 1 | 1 | 0 | 1 |
| *A. pigra* | 0 | 1 | 0 | 0 | 0 | 0 | 0 | 1 | 1 | 0 | 1 | 1 | 1 | 1 | 1 | 1 | 1 | 1 | 1 | 1 | 1 | 0 | 1 | 1 | 1 | 1 | 1 | 0 | 1 | 1 | 1 | 1 | 1 | 1 | 1 | 0 | 1 | 1 | 0 | 0 |
| *A. palliata* | 0 | 0 | 0 | 0 | 0 | 0 | 0 | 1 | 1 | 0 | 1 | 1 | 1 | 1 | 1 | 1 | 1 | 1 | 1 | 1 | 1 | 0 | 1 | 1 | 1 | 1 | 1 | 0 | 1 | 1 | 1 | 1 | 1 | 1 | 1 | 0 | 0 | 1 | 0 | 0 |
| *Lagothrix lagothricha* | 0 | 0 | 0 | 1 | 0 | 0 | 1 | 1 | 1 | 0 | 1 | 1 | 1 | 1 | 1 | 1 | 1 | 1 | 1 | 1 | 1 | 1 | 1 | 1 | 1 | 1 | 1 | 1 | 1 | 1 | 1 | 1 | 1 | 1 | 0 | 0 | 1 | 1 | 0 | 0 |
| *Cebus libidinosus* | 1 | 1 | 1 | 1 | 1 | 1 | 1 | 1 | 1 | 1 | 1 | 1 | 1 | 1 | 1 | 1 | 1 | 1 | 1 | 1 | 1 | 1 | 1 | 1 | 1 | 1 | 1 | 0 | 1 | 1 | 1 | 0 | 1 | 1 | 0 | 0 | 1 | 1 | 0 | 0 |

| *A. guariba guariba* | 0 | 0 | 0 | 0 | 0 | 0 | 0 | 0 | 0 | 0 | 0 | 0 | 0 | 0 | 0 | 0 | 0 | 0 | 0 | 0 | 0 | 0 | 0 | 0 | 0 | 0 | 0 | 0 | 1 | 0 | 1 | 0 | 0 | 0 | 0 | 0 | 0 | 0 | 0 | 0 |
| --- | --- | --- | --- | --- | --- | --- | --- | --- | --- | --- | --- | --- | --- | --- | --- | --- | --- | --- | --- | --- | --- | --- | --- | --- | --- | --- | --- | --- | --- | --- | --- | --- | --- | --- | --- | --- | --- | --- | --- | --- |
| *A. guariba clamitans* | 0 | 1 | 0 | 0 | 0 | 0 | 0 | 0 | 0 | 0 | 0 | 0 | 0 | 0 | 0 | 0 | 0 | 0 | 0 | 0 | 0 | 0 | 0 | 0 | 0 | 0 | 0 | 0 | 1 | 0 | 1 | 0 | 0 | 0 | 0 | 0 | 0 | 0 | 0 | 0 |
| *A. sara* | 0 | 0 | 0 | 0 | 0 | 0 | 1 | 0 | 1 | 0 | 0 | 0 | 0 | 0 | 1 | 0 | 0 | 0 | 1 | 0 | 0 | 1 | 1 | 1 | 1 | 1 | 0 | 0 | 1 | 0 | 1 | 0 | 0 | 0 | 0 | 0 | 0 | 0 | 1 | 0 |
| *A. macconnelli J* | 0 | 0 | 0 | 0 | 0 | 0 | 1 | 0 | 1 | 0 | 0 | 0 | 0 | 0 | 0 | 0 | 0 | 0 | 0 | 0 | 0 | 0 | 0 | 0 | 1 | 0 | 0 | 1 | 1 | 0 | 1 | 1 | 1 | 1 | 1 | 1 | 1 | 1 | 1 | 1 |
| *A. s.arctoidea* | 0 | 0 | 0 | 0 | 0 | 0 | 1 | 1 | 1 | 1 | 1 | 1 | 1 | 1 | 1 | 1 | 1 | 1 | 1 | 1 | 1 | 1 | 1 | 1 | 1 | 1 | 1 | 0 | 0 | 0 | 0 | 0 | 0 | 0 | 0 | 0 | 0 | 0 | 1 | 0 |
| *A. caraya* | 1 | 0 | 0 | 1 | 0 | 1 | 0 | 0 | 0 | 0 | 0 | 0 | 0 | 0 | 0 | 0 | 0 | 0 | 0 | 0 | 0 | 0 | 0 | 0 | 0 | 0 | 0 | 0 | 0 | 0 | 0 | 0 | 0 | 0 | 0 | 0 | 0 | 0 | 0 | 0 |
| *Alouatta belzebul* | 1 | 1 | 1 | 1 | 1 | 1 | 0 | 0 | 0 | 0 | 0 | 0 | 0 | 0 | 0 | 0 | 0 | 0 | 0 | 0 | 0 | 0 | 0 | 0 | 0 | 0 | 0 | 0 | 0 | 0 | 0 | 0 | 0 | 0 | 0 | 0 | 0 | 0 | 0 | 0 |
| *A. pigra* | 0 | 0 | 0 | 0 | 0 | 0 | 0 | 0 | 0 | 0 | 0 | 0 | 0 | 0 | 0 | 0 | 0 | 0 | 0 | 0 | 0 | 0 | 0 | 0 | 0 | 0 | 0 | 0 | 0 | 0 | 0 | 0 | 0 | 0 | 0 | 0 | 0 | 0 | 0 | 0 |
| *A. palliata* | 0 | 0 | 0 | 1 | 0 | 0 | 0 | 0 | 0 | 0 | 0 | 0 | 0 | 0 | 0 | 0 | 0 | 0 | 0 | 0 | 0 | 0 | 0 | 0 | 0 | 0 | 0 | 0 | 0 | 0 | 0 | 0 | 0 | 0 | 0 | 0 | 0 | 0 | 0 | 0 |
| *Lagothrix lagothricha* | 0 | 0 | 0 | 0 | 0 | 0 | 0 | 0 | 0 | 0 | 0 | 0 | 0 | 0 | 0 | 0 | 0 | 0 | 0 | 0 | 0 | 0 | 0 | 0 | 0 | 0 | 0 | 0 | 0 | 0 | 0 | 0 | 0 | 0 | 0 | 0 | 0 | 0 | 0 | 0 |
| *Cebus libidinosus* | 0 | 0 | 0 | 0 | 0 | 0 | 0 | 0 | 0 | 0 | 0 | 0 | 0 | 0 | 0 | 0 | 0 | 0 | 0 | 0 | 0 | 0 | 0 | 0 | 0 | 0 | 0 | 0 | 0 | 0 | 0 | 0 | 0 | 0 | 0 | 0 | 0 | 0 | 0 | 0 |

| *A. guariba guariba* | 0 | 0 | 0 | 0 | 0 | 0 | 0 | 0 | 0 | 1 | 1 | 1 | 1 | 1 | 1 | 1 | 0 | 0 |
| --- | --- | --- | --- | --- | --- | --- | --- | --- | --- | --- | --- | --- | --- | --- | --- | --- | --- | --- |
| *A. guariba clamitans* | 0 | 0 | 0 | 0 | 0 | 0 | 0 | 0 | 0 | 1 | 1 | 1 | 1 | 1 | 1 | 1 | 1 | 0 |
| *A. sara* | 0 | 0 | 0 | 1 | 1 | 1 | 1 | 1 | 1 | 0 | 0 | 0 | 0 | 0 | 0 | 0 | 0 | 0 |
| *A. macconnelli J* | 1 | 0 | 0 | 0 | 0 | 0 | 0 | 0 | 0 | 0 | 0 | 0 | 0 | 0 | 0 | 0 | 0 | 1 |
| *A. s.arctoidea* | 0 | 0 | 0 | 0 | 0 | 0 | 0 | 0 | 1 | 0 | 0 | 0 | 0 | 0 | 0 | 0 | 0 | 0 |
| *A. caraya* | 1 | 1 | 1 | 0 | 0 | 0 | 0 | 0 | 0 | 0 | 0 | 0 | 0 | 0 | 0 | 0 | 0 | 0 |
| *Alouatta belzebul* | ? | 0 | 0 | 0 | 0 | 0 | 0 | 0 | 0 | 0 | 0 | 0 | 0 | 0 | 0 | 0 | 0 | 0 |
| *A. pigra* | 0 | 0 | 0 | 0 | 0 | 0 | 0 | 0 | 0 | 0 | 0 | 0 | 0 | 0 | 0 | 0 | 0 | 0 |
| *A. palliata* | 0 | 0 | 0 | 0 | 0 | 0 | 0 | 0 | 0 | 0 | 0 | 0 | 0 | 0 | 0 | 0 | 0 | 0 |
| *Lagothrix lagothricha* | 0 | 0 | 0 | 0 | 0 | 0 | 0 | 0 | 0 | 0 | 0 | 0 | 0 | 0 | 0 | 0 | 0 | 0 |
| *Cebus libidinosus* | 0 | 0 | 0 | 0 | 0 | 0 | 0 | 0 | 0 | 0 | 0 | 0 | 0 | 0 | 0 | 0 | 0 | 0 |

-Molecular data matrix

#NEXUS

BEGIN DATA;

dimensions ntax=11 nchar=800;

format missing=?

symbols="ABCDEFGHIKLMNOPQRSTUVWXYZ"

interleave datatype=DNA gap= -;

matrix

A.g.guariba TACTCCCCGCAAAACACATCCACTAGCAAAAATCATCAACAACTCATTTA

A.g.clamitans TACTCCCCGCAAAACACATCCACTAGCAAAAATCATCAACAACTCATTTA

A.sara TACCCCCCGCAAAACACATCCACTAGCAAAAATCATTAACAACTCATTTA

A.macconneJ TACCCCCCGCAAAACACATCCACTAGCAAAAATCATTAACAACTCATTCA

A.s.arctoidea TACCCCCCGCAAAACACACCCACTAGCAAAAATCATTAACAACTCATTTA

A.caraya TACCCCCCGCAAGACACATCCACTAACAAAAATCATTAACAACTCACTCA

A.belzebul TACCCCCCGCAAAACACACCCACTAGCAAAAATCATCAACAACTCATTCA

A.pigra TACCCCCCGCAAAACTCACCCACTAGCAAAAATCATCAACAATTCATTCA

A.palliata TACCCCCCGCAAAACTCACCCACTAGCAAAAATCATCAACAATTCATTCA

L.lagothricha CACCCCTCGCAA-ACACACCCACTAGCAAAAATCATTAACAACTCACTCA

C.libidinosus CTCTTCCCGCAAAACACATCCACTAATAAAAATTATTAATAACTCACTTT

A.g.guariba TTGATCTCCCTACACCATCCAACATCTCCGCCTGATGAAATTTCGGCTCA

A.g.clamitans TTGATCTCCCTACACCATCCAACATCTCCGCCTGATGAAATTTCGGCTCA

A.sara TTGATCTTCCCACACCATCCAACATCTCCGCCTGATGAAACTTCGGCTCA

A.macconneJ TTGATCTCCCCACACCATCCAACATCTCCGCCTGATGAAATTTCGGCTCA

A.s.arctoidea TTGACCTTCCCACACCATCCAACATCTCCGCTTGATGAAATTTCGGCTCG

A.caraya TTGATCTCCCCACACCATCCAACATTTCCGCCTGATGAAATTTCGGCTCA

A.belzebul TTGACCTTCCTACACCATCCAACATCTCCGCCTGATGAAATTTTGGCTCA

A.pigra TTGACCTCCCTACACCATCCAACATCTCCGCCTGATGAAATTTCGGCTCA

A.palliata TTGACCTCCCTACACCATCCAACATCTCCGCCTGGTGAAATTTCGGCTCA

L.lagothricha TTGACCTACCCTCACCATCCAATATTTCTGCTTGATGAAATTTTGGTTCA

C.libidinosus TTGACCTCCCTACACCATCCAACATCTCCTCCTGATGAAACTTCGGATCA

A.g.guariba CTCCTAGGTATTTGCCTAATTATTCAAATCACTACAGGTCTATTCTTAGC

A.g.clamitans CTCCTAGGTATTTGCCTAATTATTCAAATCACTACAGGTCTATTCTTAGC

A.sara CTTCTAGGTATTTGCCTAATTATCCAAATCACTACAGGCCTATTCTTAGC

A.macconneJ CTCCTAGGTATTTGCCTGATTATCCAAATTACTACAGGTCTATTCTTAGC

A.s.arctoidea CTCCTAGGTATCTGCCTGATTATCCAAATCACTACAGGTCTATTCTTAGC

A.caraya CTCCTAGGTATTTGCCTAATTATCCAAATCACTACAGGTCTATTCCTAGC

A.belzebul CTCCTAGGTATTTGCCTAATTATTCAAATCACCACAGGTCTATTCTTAGC

A.pigra CTCCTAGGCATTTGCCTCATTATTCAAATTACTACAGGCCTATTCTTAGC

A.palliata CTCCTAGGTATTTGCCTAATTATTCAAATCACTACAGGTCTATTCTTAGC

L.lagothricha CTCTTAGGCATTTGTTTAATTATTCAAATCGCCACAGGCCTATTCCTAGC

C.libidinosus CTTCTAGGCGCCTGCCTAATAATTCAAATCACCACAGGCCTATTCTTAGC

A.g.guariba CATACACTATACACCAGACACTTCAACTGCCTTCTCCTCAGTCGCCCACA

A.g.clamitans CATACACTATACACCAGACACTTCAACTGCCTTCTCCTCAGTCGCCCACA

A.sara CATACACTACACACCAGACACCTCAACTGCCTTCTCCTCAGTCGCCCACA

A.macconneJ CATACACTACACACCAGACACCTCAACTGCCTTCTCCTCAGTCGCCCACA

A.s.arctoidea TATGCACTACACACCAGACACCTCAACTGCCTTCTCCTCAGTCGCCCACA

A.caraya CATACATTATACACCAGACACTTCAACTGCCTTCTCCTCGGTCGCCCACA

A.belzebul CATACATTATACACCAGACACTTCAACTGCCTTCTCCTCAGTTACCCATA

A.pigra CATACACTATACACCAGATACTTCAACCGCCTTCTCTTCAGTCGCTCACA

A.palliata CATACACTATACACCAGACACTTCAACCGCCTTCTCCTCAGTCGCCCACA

L.lagothricha CATACACTATACACCAGACACTTCAACCGCCTTCTCTTCAGTTGCCCATA

C.libidinosus AATACACTACACGCCAGACACCTCAACCGCCTTCTCCTCAGTAGCTCACA

A.g.guariba TCACCCGAGACGTCAACTACGGCTGAATAATCCGCTACCTACACGCCAAT

A.g.clamitans TCACCCGAGACGTCAACTACGGCTGAATAATCCGCTACCTACACGCCAAT

A.sara TCACCCGAGACGTCAACTACGGCTGAATAATCCGCTACCTACACGCCAAT

A.macconneJ TCACCCGAGACGTCAACTACGGCTGAATAATCCGCTACCTACACGCCAAT

A.s.arctoidea TCACCCGAGACGTCAACTACGGCTGAATAATCCGCTACCTACACGCCAAC

A.caraya TCACCCGAGACGTCAACTACGGCTGAATAATCCGCTACCTACACGCCAAC

A.belzebul TCACCCGAGACGTCAATTACGGCTGAATAATCCGCTACCTACACGCCAAT

A.pigra TCACCCGAGACGTCAACTATGGCTGAATAATCCGCTACCTACACGCCAAT

A.palliata TCACCCGAGACGTCAACTATGGCTGAATAATCCGCTATCTACATGCCAAC

L.lagothricha TTGCCCGAGACGTAAACTACGGATGAATAATCCGCTACCTACACGCCAAC

C.libidinosus TCACCCGAGATATCAACTATGGCTGAATAATCCGCCTCCTACACGCCAAT

A.g.guariba GGCGCCTCCATATTCTTCATCTGCCTCTTCCTTCACATTGGCCGAGGCCT

A.g.clamitans GGCGCCTCCATATTCTTCATCTGCCTCTTCCTTCACATTGGCCGAGGCCT

A.sara GGCGCCTCCATATTCTTCATCTGCCTCTTCCTCCACATTGGCCGAGGCTT

A.macconneJ GGCGCCTCCATATTCTTCATCTGCCTCTTCCTCCACATTGGCCGAGGCTT

A.s.arctoidea GGCGCCTCCATATTTTTCATCTGCCTCTTCCTCCACATTGGCCGAGGCTT

A.caraya GGCGCTTCCATATTCTTCATCTGCCTATTCCTCCACATTGGCCGAGGCTT

A.belzebul GGCGCCTCCATATTCTTTATCTGCCTCTTTCTCCACATTGGCCGAGGCTT

A.pigra GGCGCCTCCATATTCTTTATCTGTCTCTTTCTCCACATTGGCCGAGGCTT

A.palliata GGCGCCTCCATATTCTTTATCTGCCTCTTTCTCCACATTGGCCGAGGCTT

L.lagothricha GGTGCTTCCATATTCTTTATTTGCCTCTTCCTACACGTCGGCCGAGGCTT

C.libidinosus GGTGCCTCCGTATTTTTTGCATGCTTATTCCTCCACATCGGCCGAGGCCT

A.g.guariba ATATTATGGATCATTCCTTTTTCTGAAGACCTGAAACATCGGTATTATCC

A.g.clamitans ATATTATGGATCATTCCTTTTTCTGAAGACCTGAAACGTCGGTATTATCC

A.sara ATATTACGGATCATTCCTTTTTCTGAAGACCTGAAACGTCGGTATTTTCC

A.macconneJ ATATTATGGATCATTCCTTTTTCTGAAGACCTGAAACGTCGGTATTATCC

A.s.arctoidea ATATTACGGATCATTCCTTTTTCTGAAGACCTGAAACGTCGGTATTATCC

A.caraya ATATTACGGGTCATTCCTTTTTCTGAAGACCTGAAACGTCGGTATTATCC

A.belzebul ATATTACGGATCATTCCTTTTTCTGAAGACCTGAAACGTCGGTATTATCC

A.pigra ATATTACGGATCATTCCTTTTTCTGGAGACCTGAAACATCGGTATTATCC

A.palliata ATATTACGGATCATTCCTTTTTCTGGAGACCTGGAACGTCGGTATTATCC

L.lagothricha ATATTATGGATCCTTCCTTTCTCTGGAGACTTGAAACGTAGGTATTATTC

C.libidinosus CTACTACGGATCCTTTCTCCTTCTAAAGACCTGAAACATCGGTACAATCC

A.g.guariba TTCTACTCACAACTATAGCCACAGCATTCATAGGCTATGTCCTCCCATGA

A.g.clamitans TTCTACTCACAACTATAGCCACAGCATTCATAGGCTATGTCCTCCCATGA

A.sara TCCTACTCACAACCATAGCTACAGCATTCATAGGCTATGTCCTCCCATGA

A.macconneJ TCCTACTCACAACCATAGCCACAGCATTCATAGGCTATGTCCTCCCATGG

A.s.arctoidea TCCTACTCACAACCATAGCCACAGCATTCATAGGCTATGTCCTCCCATGA

A.caraya TCCTACTCACAACCATAGCCACAGCATTCATAGGCTACGTCCTCCCATGA

A.belzebul TCCTACTCACAACTATAGCCACAGCATTCATAGGCTATGTCCTCCCATGA

A.pigra TCCTACTCACAACCATAGCCACAGCATTCATAGGCTATGTCCTCCCATGA

A.palliata TCCTACTCACAACCATAGCCACAGCATTCATAGGCTATGTCCTCCCATGA

L.lagothricha TACTACTTACAACCATAGCCACAGCATTCATAGGTTACGTCCTCCCATGG

C.libidinosus TACTATTAATAACAATAGCCACAGCCTTTATAGGCTACGTATTGCCGTGG

A.g.guariba GGCCAAATATCATTCTGAGGGGCCACAGTAATTACAAACCTTCTATCAGC

A.g.clamitans GGCCAAATATCATTCTGAGGGGCCACAGTAATTACAAACCTCCTATCAGC

A.sara GGCCAAATATCATTCTGAGGGGCCACAGTAATTACAAACCTTCTATCAGC

A.macconneJ GGCCAAATATCATTCTGAGGGGCCACAGTAATTACAAACCTTCTATCAGC

A.s.arctoidea GGCCAAATATCATTCTGAGGTGCCACAGTAATTACAAACCTCCTATCAGC

A.caraya GGCCAAATATCATTCTGAGGGGCCACAGTAATTACAAACCTTCTATCAGC

A.belzebul GGCCAAATATCATTCTGAGGGGCCACAGTAATTACAAATCTTCTATCAGC

A.pigra GGCCAAATATCATTCTGAGGCGCCACAGTAATTACAAACCTTCTGTCAGC

A.palliata GGCCAAATATCATTCTGAGGGGCCACAGTAATTACAAACCTTCTGTCAGC

L.lagothricha GGCCAAATATCATTCTGAGGGGCTACAGTAATCACAAATCTTCTATCAGC

C.libidinosus GGCCAAATATCATTCTGAGGGGCCACAGTTATTACAAACCTTTTATCAGC

A.g.guariba CATCCCATACATCGGATCTGACCTCGTACAATGAATCTGGGGTGGTTTCT

A.g.clamitans CATCCCATACATCGGATCTGACCTCGTACAATGAATCTGAGGTGGTTTCT

A.sara CATCCCATACATCGGATCTGACCTCGTACAATGAATCTGAGGTGGCTTCT

A.macconneJ CATCCCATACATCGGATCTGACCTCGTACAATGAATCTGAGGCGGCTTCT

A.s.arctoidea CATCCCATACATCGGATCTGACCTCGTACAATGAATCTGAGGTGGCTTCT

A.caraya CATCCCATACATCGGATCTGACCTCGTACAATGAATCTGAGGTGGGTTCT

A.belzebul CATCCCATACATCGGATCTGATCTCGTACAATGAATCTGAGGTGGTTTCT

A.pigra CATCCCATATATCGGATCTGACCTTGTACAATGAATCTGAGGTGGCTTCT

A.palliata CATTCCATATATCGGGTCTGACCTCGTACAATGAATCTGAGGTGGCTTCT

L.lagothricha CATCCCCTATATTGGGTCCAGTCTTGTAGAGTGAATCTGAGGTGGTTTCT

C.libidinosus CATCCCCTATACCGGACATGACCTTGTACAATGAATCTGAGGTGGCTTTT

A.g.guariba CAGTAGATAAAGCCACCCTCACACGATTTTTCACCTTTCACTTCATTCTA

A.g.clamitans CAGTAGATAAAGCCACCCTCACACGATTTTTCACCTTTCACTTCATTCTA

A.sara CAGTAGATAAAGCCACCCTCACACGATTTTTCACCTTTCACTTTATTCTA

A.macconneJ CAGTAGATAAAGCCACCCTCACACGATTTTTCACCTTTCACTTTATTCTA

A.s.arctoidea CAGTAGATAAAGCTACCCTCACACGATTTTTCACCTTCCACTTTATCTTA

A.caraya CAGTAGATAAAGCCACCCTTACACGATTTTTCACCTTTCACTTTATTTTA

A.belzebul CAGTAGATAAAGCCACCCTCACACGATTTTTCACCTTTCACTTTATTCTA

A.pigra CAGTAGATAAAGCCACCCTCACACGATTTTTCACCTTTCACTTTATTTTA

A.palliata CAGTAGATAAAGCCACCCTCACACGATTTTTCACCTTTCACTTTATCTTG

L.lagothricha CAGTAGACAAAGCCACCCTTACACGATTCTTTACTTTCCACTTTATCTTA

C.libidinosus CAGTGGATAAGCCCACCCTCACACGATTCTTTACCTTTCACTTTATTTTA

A.g.guariba CCCTTTATTATCGCTGCCCTAGCAACCATCCATCTCTTGTTTCTGCATGA

A.g.clamitans CCCTTTATTATCGCTGCCCTAGCAACCATCCACCTCTTGTTTCTGCATGA

A.sara CCCTTTATCATCGCTGCCCTAGCAACCATCCACCTCTTGTTTCTGCATGA

A.macconneJ CCCTTTATCATCGCTGCCCTGGCAACCATCCACCTCTTGTTTCTGCATGA

A.s.arctoidea CCCTTTATTATCGCTGCCCTAGCAACCATCCACCTCTTGTTTCTGCATGA

A.caraya CCCTTTATCATTGCTGCCCTAGCAACTATCCACCTCTTGTTTCTGCATGA

A.belzebul CCCTTTATTATCGCTGCCCTAGCAACCATTCACCTCTTGTTTCTGCATGA

A.pigra CCATTTATTATCGCTGCCCTAGCAACCATCCATCTCTTGTTTCTGCATGA

A.palliata CCATTTATTATCGCTGCCCTAGCAACCATCCACCTCTTGTTTCTGCATGA

L.lagothricha CCCTTTATTATTGCAGCCCTAGCAACTATTCACCTATTATTTCTGCATGA

C.libidinosus CCTTTCATTATCACAGCTCTAACAACCATTCACCTCTTATTTCTGCATGA

A.g.guariba AACAGGATCAAGTAACCCATCAGGAATGACATCGGACCTCGACAAAATCA

A.g.clamitans AACAGGATCAAGTAACCCATCAGGAATGACATCGGACCTTGACAAAATCA

A.sara AACTGGATCAAGTAACCCATCAGGAATAGCATCAGACCCCGATAAAATCA

A.macconneJ AACAGGATCAAGTAATCCATCAGGAGTAGCATCAGACCTCGATAAAATCA

A.s.arctoidea AACAGGATCAAGTAACCCATCAGGAATGACATCAGACCTCGACAAAATCA

A.caraya GACAGGATCAAGTAACCCATCAGGAATGGCATCAGACCTCGACAAAATCA

A.belzebul AACAGGATCAAGTAACCCATCAGGAATAACATCAGACCTCGATAAAATCA

A.pigra AACAGGATCAAGTAACCCGTCAGGAATGGCATCAGACCTCGACAAAATTA

A.palliata AACAGGATCAAGTAACCCATCAGGAGTAGCATCGGACCTCGACAAAATTA

L.lagothricha CACAGGGTCAAGTAATCCATCAGGAATAACATCAGACCCAGATAAAATCA

C.libidinosus AACAGGCTCAAATAACCCATCAGGAATAACATCCAACCCCGATAAAATTA

A.g.guariba CATTTCACCCCTACTATACAACCAAAGATATCCTAGGCCTAATTTTCCTC

A.g.clamitans CATTTCACCCCTACTATACAACCAAAGATATCCTAGGCCTAACTATTCTC

A.sara CATTTCACCCTTATTATACAACCAAAGACATCCTAGGTCTAATCATTCTC

A.macconneJ CATTTCACCCCTATTATACAATCAAAGACATCCTAGGTCTAATTATTCTC

A.s.arctoidea CATTTCACCCCTATTATACAACCAAAGACATTCTAGGCCTAATCATTCTC

A.caraya CATTTCACCCCTACTATACAACCAAAGACATCCTAGGCCTAATTATTCTC

A.belzebul CATTTCACCCCTACTATACAACCAAGGACATCCTAGGCCTAATTATTCTC

A.pigra CATTTCACCCCTACTATACAACCAAAGACATCCTAGGCTTAATTATTCTC

A.palliata CATTTCACCCCTACTATACAACCAAAGATATCCTAGGCTTAATTATTCTC

L.lagothricha CATTCCACCCCTACTATACAATCAAGGACATTTTTGGTTTAATTCTTCTT

C.libidinosus CATTCCATCCCTATTACACAACCAAAGACATTTTTGGACTAACCCTTCTT

A.g.guariba CTCCTATGTCTAACAAGCCTGACCCTATTTTCACCTGACCTTCTAACCGA

A.g.clamitans CTCCTATGTCTAATAAGCCTGACCCTATTTTCACCTGACCTTCTAACCGA

A.sara CTCCTATGCCTAATAAGCCTAACCCTATTTTCACCTGACCTTCTAACCGA

A.macconneJ CTCCTATGCCTAATAAGCCTAACCCTATTTTCACCTGACCTTCTAACTGA

A.s.arctoidea CTCCTATGCCTAGTAAGCCTAACCCTATTTTCACCTGACCTTCTAACTGA

A.caraya CTCCTATGTCTAATAAGCCTAACCCTATTTTCACCTGACCTTCTAACCGA

A.belzebul CTCCTATGTCTAATAAGCCTAACCCTATTTTCACCTGACCTTCTAACCGA

A.pigra CTTCTATGCCTAATGAGCCTAACTCTATTTTCACCCGACCTTTTAACCGA

A.palliata CTCCTATGCCTAATAAGCCTAACCCTATTTCTACCCGACCTTCTAACCGA

L.lagothricha CTCCTATGCTTAATAAACCTAACCTTATTCTCACCTGACCTCTTA-----

C.libidinosus CTCTTGCTCCTCATAAACCTAACCCTATTTACTCCTGACCTTTTAATCGA

A.g.guariba CCCAGATAATTATACACTAGCTAATCCCCTCAACACTCCACCCCA-----

A.g.clamitans CCCAGATAATTATACACTAGCTAATCCCCTCAACACTCCACCCCACATTA

A.sara CCCAGATAATTACACACTAGCTAACCCCCTCAACACCCCACCCCA-----

A.macconneJ CCCAGATAATTACACACTAGCTAATCCCCTCAACACTCCACCCCA-----

A.s.arctoidea CCCAGATAATTATACACTAGCTAACCCCCTCAACACCCCACCCCA-----

A.caraya CCCAGATAATTACACACTAGCTAACCCCCTCAACACCCCACCCCACATTA

A.belzebul CCCAGATAATTATACACTAGCTAATCCCCTCAACACCCCGCCCCACATTA

A.pigra CCCAGACAATTATACACTAGCTAATCCCCTCAACACCCCACCTCA-----

A.palliata CCCAGACAATTATACACTAGCCAACCCTCTCAACACCCCACCTCA-----

L.lagothricha --------------------------------------------------

C.libidinosus CCCAGACAACTTCACACTAGCTAACCCCCTGAATACTCCACCTCATATTA

;

end;

-Combined data matrix

#Nexus

Begin data;

dimensions ntax=11 nchar=899;

format datatype=standard interleave symbols="01ACGT" gap=-;

| *A. guariba guariba* | 0 | 0 | 0 | 0 | 0 | 1 | 0 | 1 | 1 | 0 | 1 | 1 | 1 | 1 | 1 | 1 | 1 | 1 | 1 | 1 | 1 | 1 | 1 | 1 | 1 | 1 | 1 | 1 | 1 | 1 | 1 | 1 | 1 | 1 | 0 | 1 | 1 | 1 | 1 | 0 |
| --- | --- | --- | --- | --- | --- | --- | --- | --- | --- | --- | --- | --- | --- | --- | --- | --- | --- | --- | --- | --- | --- | --- | --- | --- | --- | --- | --- | --- | --- | --- | --- | --- | --- | --- | --- | --- | --- | --- | --- | --- |
| *A. guariba clamitans* | 0 | 0 | 0 | 0 | 0 | 1 | 0 | 1 | 1 | 0 | 1 | 1 | 1 | 1 | 1 | 1 | 1 | 1 | 1 | 1 | 1 | 1 | 1 | 1 | 1 | 1 | 1 | 1 | 1 | 1 | 1 | 1 | 1 | 1 | 0 | 1 | 1 | 1 | 1 | 0 |
| *A. sara* | 0 | 0 | 0 | 0 | 0 | 0 | 0 | 1 | 1 | 0 | 1 | 1 | 1 | 1 | 1 | 1 | 1 | 1 | 1 | 1 | 1 | 1 | 1 | 1 | 1 | 1 | 1 | 1 | 1 | 1 | 1 | 1 | 1 | 0 | 0 | 1 | 1 | 1 | 1 | 0 |
| *A. macconnelli J* | 0 | 0 | 0 | 0 | 0 | 0 | 0 | 1 | 1 | 0 | 1 | 1 | 1 | 1 | 1 | 1 | 1 | 1 | 1 | 1 | 1 | 1 | 1 | 1 | 1 | 1 | 1 | 1 | 1 | 1 | 1 | 1 | 1 | 1 | 0 | 1 | 1 | 1 | 1 | 0 |
| *A. s.arctoidea* | 0 | 0 | 0 | 0 | 0 | 0 | 0 | 1 | 0 | 0 | 1 | 1 | 1 | 1 | 1 | 1 | 1 | 1 | 1 | 1 | 1 | 1 | 1 | 1 | 1 | 1 | 1 | 1 | 1 | 1 | 1 | 1 | 1 | 0 | 1 | 1 | 1 | 1 | 1 | 0 |
| *A. caraya* | 0 | 0 | 0 | 0 | 0 | 0 | 0 | 1 | 1 | 0 | 1 | 1 | 1 | 1 | 1 | 1 | 1 | 1 | 1 | 1 | 1 | 1 | 1 | 1 | 1 | 1 | 1 | 1 | 1 | 1 | 1 | 1 | 1 | 1 | 1 | 1 | 1 | 1 | 1 | 0 |
| *Alouatta belzebul* | 0 | 0 | 0 | 0 | 0 | 0 | 0 | 1 | 1 | 0 | 1 | 1 | 1 | 1 | 1 | 1 | 1 | 1 | 1 | 1 | 1 | 1 | 1 | 1 | 1 | 1 | 1 | 1 | 1 | 1 | 1 | 1 | 1 | 1 | 1 | ? | 1 | 1 | 0 | 1 |
| *A. pigra* | 0 | 1 | 0 | 0 | 0 | 0 | 0 | 1 | 1 | 0 | 1 | 1 | 1 | 1 | 1 | 1 | 1 | 1 | 1 | 1 | 1 | 0 | 1 | 1 | 1 | 1 | 1 | 0 | 1 | 1 | 1 | 1 | 1 | 1 | 1 | 0 | 1 | 1 | 0 | 0 |
| *A. palliata* | 0 | 0 | 0 | 0 | 0 | 0 | 0 | 1 | 1 | 0 | 1 | 1 | 1 | 1 | 1 | 1 | 1 | 1 | 1 | 1 | 1 | 0 | 1 | 1 | 1 | 1 | 1 | 0 | 1 | 1 | 1 | 1 | 1 | 1 | 1 | 0 | 0 | 1 | 0 | 0 |
| *Lagothrix lagothricha* | 0 | 0 | 0 | 1 | 0 | 0 | 1 | 1 | 1 | 0 | 1 | 1 | 1 | 1 | 1 | 1 | 1 | 1 | 1 | 1 | 1 | 1 | 1 | 1 | 1 | 1 | 1 | 1 | 1 | 1 | 1 | 1 | 1 | 1 | 0 | 0 | 1 | 1 | 0 | 0 |
| *Cebus libidinosus* | 1 | 1 | 1 | 1 | 1 | 1 | 1 | 1 | 1 | 1 | 1 | 1 | 1 | 1 | 1 | 1 | 1 | 1 | 1 | 1 | 1 | 1 | 1 | 1 | 1 | 1 | 1 | 0 | 1 | 1 | 1 | 0 | 1 | 1 | 0 | 0 | 1 | 1 | 0 | 0 |

| *A. guariba guariba* | 0 | 0 | 0 | 0 | 0 | 0 | 0 | 0 | 0 | 0 | 0 | 0 | 0 | 0 | 0 | 0 | 0 | 0 | 0 | 0 | 0 | 0 | 0 | 0 | 0 | 0 | 0 | 0 | 1 | 0 | 1 | 0 | 0 | 0 | 0 | 0 | 0 | 0 | 0 | 0 |
| --- | --- | --- | --- | --- | --- | --- | --- | --- | --- | --- | --- | --- | --- | --- | --- | --- | --- | --- | --- | --- | --- | --- | --- | --- | --- | --- | --- | --- | --- | --- | --- | --- | --- | --- | --- | --- | --- | --- | --- | --- |
| *A. guariba clamitans* | 0 | 1 | 0 | 0 | 0 | 0 | 0 | 0 | 0 | 0 | 0 | 0 | 0 | 0 | 0 | 0 | 0 | 0 | 0 | 0 | 0 | 0 | 0 | 0 | 0 | 0 | 0 | 0 | 1 | 0 | 1 | 0 | 0 | 0 | 0 | 0 | 0 | 0 | 0 | 0 |
| *A. sara* | 0 | 0 | 0 | 0 | 0 | 0 | 1 | 0 | 1 | 0 | 0 | 0 | 0 | 0 | 1 | 0 | 0 | 0 | 1 | 0 | 0 | 1 | 1 | 1 | 1 | 1 | 0 | 0 | 1 | 0 | 1 | 0 | 0 | 0 | 0 | 0 | 0 | 0 | 1 | 0 |
| *A. macconnelli J* | 0 | 0 | 0 | 0 | 0 | 0 | 1 | 0 | 1 | 0 | 0 | 0 | 0 | 0 | 0 | 0 | 0 | 0 | 0 | 0 | 0 | 0 | 0 | 0 | 1 | 0 | 0 | 1 | 1 | 0 | 1 | 1 | 1 | 1 | 1 | 1 | 1 | 1 | 1 | 1 |
| *A. s.arctoidea* | 0 | 0 | 0 | 0 | 0 | 0 | 1 | 1 | 1 | 1 | 1 | 1 | 1 | 1 | 1 | 1 | 1 | 1 | 1 | 1 | 1 | 1 | 1 | 1 | 1 | 1 | 1 | 0 | 0 | 0 | 0 | 0 | 0 | 0 | 0 | 0 | 0 | 0 | 1 | 0 |
| *A. caraya* | 1 | 0 | 0 | 1 | 0 | 1 | 0 | 0 | 0 | 0 | 0 | 0 | 0 | 0 | 0 | 0 | 0 | 0 | 0 | 0 | 0 | 0 | 0 | 0 | 0 | 0 | 0 | 0 | 0 | 0 | 0 | 0 | 0 | 0 | 0 | 0 | 0 | 0 | 0 | 0 |
| *Alouatta belzebul* | 1 | 1 | 1 | 1 | 1 | 1 | 0 | 0 | 0 | 0 | 0 | 0 | 0 | 0 | 0 | 0 | 0 | 0 | 0 | 0 | 0 | 0 | 0 | 0 | 0 | 0 | 0 | 0 | 0 | 0 | 0 | 0 | 0 | 0 | 0 | 0 | 0 | 0 | 0 | 0 |
| *A. pigra* | 0 | 0 | 0 | 0 | 0 | 0 | 0 | 0 | 0 | 0 | 0 | 0 | 0 | 0 | 0 | 0 | 0 | 0 | 0 | 0 | 0 | 0 | 0 | 0 | 0 | 0 | 0 | 0 | 0 | 0 | 0 | 0 | 0 | 0 | 0 | 0 | 0 | 0 | 0 | 0 |
| *A. palliata* | 0 | 0 | 0 | 1 | 0 | 0 | 0 | 0 | 0 | 0 | 0 | 0 | 0 | 0 | 0 | 0 | 0 | 0 | 0 | 0 | 0 | 0 | 0 | 0 | 0 | 0 | 0 | 0 | 0 | 0 | 0 | 0 | 0 | 0 | 0 | 0 | 0 | 0 | 0 | 0 |
| *Lagothrix lagothricha* | 0 | 0 | 0 | 0 | 0 | 0 | 0 | 0 | 0 | 0 | 0 | 0 | 0 | 0 | 0 | 0 | 0 | 0 | 0 | 0 | 0 | 0 | 0 | 0 | 0 | 0 | 0 | 0 | 0 | 0 | 0 | 0 | 0 | 0 | 0 | 0 | 0 | 0 | 0 | 0 |
| *Cebus libidinosus* | 0 | 0 | 0 | 0 | 0 | 0 | 0 | 0 | 0 | 0 | 0 | 0 | 0 | 0 | 0 | 0 | 0 | 0 | 0 | 0 | 0 | 0 | 0 | 0 | 0 | 0 | 0 | 0 | 0 | 0 | 0 | 0 | 0 | 0 | 0 | 0 | 0 | 0 | 0 | 0 |

| *A. guariba guariba* | 0 | 0 | 0 | 0 | 0 | 0 | 0 | 0 | 0 | 1 | 1 | 1 | 1 | 1 | 1 | 1 | 0 | 0 |
| --- | --- | --- | --- | --- | --- | --- | --- | --- | --- | --- | --- | --- | --- | --- | --- | --- | --- | --- |
| *A. guariba clamitans* | 0 | 0 | 0 | 0 | 0 | 0 | 0 | 0 | 0 | 1 | 1 | 1 | 1 | 1 | 1 | 1 | 1 | 0 |
| *A. sara* | 0 | 0 | 0 | 1 | 1 | 1 | 1 | 1 | 1 | 0 | 0 | 0 | 0 | 0 | 0 | 0 | 0 | 0 |
| *A. macconnelli J* | 1 | 0 | 0 | 0 | 0 | 0 | 0 | 0 | 0 | 0 | 0 | 0 | 0 | 0 | 0 | 0 | 0 | 1 |
| *A. s.arctoidea* | 0 | 0 | 0 | 0 | 0 | 0 | 0 | 0 | 1 | 0 | 0 | 0 | 0 | 0 | 0 | 0 | 0 | 0 |
| *A. caraya* | 1 | 1 | 1 | 0 | 0 | 0 | 0 | 0 | 0 | 0 | 0 | 0 | 0 | 0 | 0 | 0 | 0 | 0 |
| *Alouatta belzebul* | ? | 0 | 0 | 0 | 0 | 0 | 0 | 0 | 0 | 0 | 0 | 0 | 0 | 0 | 0 | 0 | 0 | 0 |
| *A. pigra* | 0 | 0 | 0 | 0 | 0 | 0 | 0 | 0 | 0 | 0 | 0 | 0 | 0 | 0 | 0 | 0 | 0 | 0 |
| *A. palliata* | 0 | 0 | 0 | 0 | 0 | 0 | 0 | 0 | 0 | 0 | 0 | 0 | 0 | 0 | 0 | 0 | 0 | 0 |
| *Lagothrix lagothricha* | 0 | 0 | 0 | 0 | 0 | 0 | 0 | 0 | 0 | 0 | 0 | 0 | 0 | 0 | 0 | 0 | 0 | 0 |
| *Cebus libidinosus* | 0 | 0 | 0 | 0 | 0 | 0 | 0 | 0 | 0 | 0 | 0 | 0 | 0 | 0 | 0 | 0 | 0 | 0 |

A.g.guariba TACTCCCCGCAAAACACATCCACTAGCAAAAATCATCAACAACTCATTTA

A.g.clamitans TACTCCCCGCAAAACACATCCACTAGCAAAAATCATCAACAACTCATTTA

A.sara TACCCCCCGCAAAACACATCCACTAGCAAAAATCATTAACAACTCATTTA

A.macconneJ TACCCCCCGCAAAACACATCCACTAGCAAAAATCATTAACAACTCATTCA

A.s.arctoidea TACCCCCCGCAAAACACACCCACTAGCAAAAATCATTAACAACTCATTTA

A.caraya TACCCCCCGCAAGACACATCCACTAACAAAAATCATTAACAACTCACTCA

A.belzebul TACCCCCCGCAAAACACACCCACTAGCAAAAATCATCAACAACTCATTCA

A.pigra TACCCCCCGCAAAACTCACCCACTAGCAAAAATCATCAACAATTCATTCA

A.palliata TACCCCCCGCAAAACTCACCCACTAGCAAAAATCATCAACAATTCATTCA

L.lagothricha CACCCCTCGCAA-ACACACCCACTAGCAAAAATCATTAACAACTCACTCA

C.libidinosus CTCTTCCCGCAAAACACATCCACTAATAAAAATTATTAATAACTCACTTT

A.g.guariba TTGATCTCCCTACACCATCCAACATCTCCGCCTGATGAAATTTCGGCTCA

A.g.clamitans TTGATCTCCCTACACCATCCAACATCTCCGCCTGATGAAATTTCGGCTCA

A.sara TTGATCTTCCCACACCATCCAACATCTCCGCCTGATGAAACTTCGGCTCA

A.macconneJ TTGATCTCCCCACACCATCCAACATCTCCGCCTGATGAAATTTCGGCTCA

A.s.arctoidea TTGACCTTCCCACACCATCCAACATCTCCGCTTGATGAAATTTCGGCTCG

A.caraya TTGATCTCCCCACACCATCCAACATTTCCGCCTGATGAAATTTCGGCTCA

A.belzebul TTGACCTTCCTACACCATCCAACATCTCCGCCTGATGAAATTTTGGCTCA

A.pigra TTGACCTCCCTACACCATCCAACATCTCCGCCTGATGAAATTTCGGCTCA

A.palliata TTGACCTCCCTACACCATCCAACATCTCCGCCTGGTGAAATTTCGGCTCA

L.lagothricha TTGACCTACCCTCACCATCCAATATTTCTGCTTGATGAAATTTTGGTTCA

C.libidinosus TTGACCTCCCTACACCATCCAACATCTCCTCCTGATGAAACTTCGGATCA

A.g.guariba CTCCTAGGTATTTGCCTAATTATTCAAATCACTACAGGTCTATTCTTAGC

A.g.clamitans CTCCTAGGTATTTGCCTAATTATTCAAATCACTACAGGTCTATTCTTAGC

A.sara CTTCTAGGTATTTGCCTAATTATCCAAATCACTACAGGCCTATTCTTAGC

A.macconneJ CTCCTAGGTATTTGCCTGATTATCCAAATTACTACAGGTCTATTCTTAGC

A.s.arctoidea CTCCTAGGTATCTGCCTGATTATCCAAATCACTACAGGTCTATTCTTAGC

A.caraya CTCCTAGGTATTTGCCTAATTATCCAAATCACTACAGGTCTATTCCTAGC

A.belzebul CTCCTAGGTATTTGCCTAATTATTCAAATCACCACAGGTCTATTCTTAGC

A.pigra CTCCTAGGCATTTGCCTCATTATTCAAATTACTACAGGCCTATTCTTAGC

A.palliata CTCCTAGGTATTTGCCTAATTATTCAAATCACTACAGGTCTATTCTTAGC

L.lagothricha CTCTTAGGCATTTGTTTAATTATTCAAATCGCCACAGGCCTATTCCTAGC

C.libidinosus CTTCTAGGCGCCTGCCTAATAATTCAAATCACCACAGGCCTATTCTTAGC

A.g.guariba CATACACTATACACCAGACACTTCAACTGCCTTCTCCTCAGTCGCCCACA

A.g.clamitans CATACACTATACACCAGACACTTCAACTGCCTTCTCCTCAGTCGCCCACA

A.sara CATACACTACACACCAGACACCTCAACTGCCTTCTCCTCAGTCGCCCACA

A.macconneJ CATACACTACACACCAGACACCTCAACTGCCTTCTCCTCAGTCGCCCACA

A.s.arctoidea TATGCACTACACACCAGACACCTCAACTGCCTTCTCCTCAGTCGCCCACA

A.caraya CATACATTATACACCAGACACTTCAACTGCCTTCTCCTCGGTCGCCCACA

A.belzebul CATACATTATACACCAGACACTTCAACTGCCTTCTCCTCAGTTACCCATA

A.pigra CATACACTATACACCAGATACTTCAACCGCCTTCTCTTCAGTCGCTCACA

A.palliata CATACACTATACACCAGACACTTCAACCGCCTTCTCCTCAGTCGCCCACA

L.lagothricha CATACACTATACACCAGACACTTCAACCGCCTTCTCTTCAGTTGCCCATA

C.libidinosus AATACACTACACGCCAGACACCTCAACCGCCTTCTCCTCAGTAGCTCACA

A.g.guariba TCACCCGAGACGTCAACTACGGCTGAATAATCCGCTACCTACACGCCAAT

A.g.clamitans TCACCCGAGACGTCAACTACGGCTGAATAATCCGCTACCTACACGCCAAT

A.sara TCACCCGAGACGTCAACTACGGCTGAATAATCCGCTACCTACACGCCAAT

A.macconneJ TCACCCGAGACGTCAACTACGGCTGAATAATCCGCTACCTACACGCCAAT

A.s.arctoidea TCACCCGAGACGTCAACTACGGCTGAATAATCCGCTACCTACACGCCAAC

A.caraya TCACCCGAGACGTCAACTACGGCTGAATAATCCGCTACCTACACGCCAAC

A.belzebul TCACCCGAGACGTCAATTACGGCTGAATAATCCGCTACCTACACGCCAAT

A.pigra TCACCCGAGACGTCAACTATGGCTGAATAATCCGCTACCTACACGCCAAT

A.palliata TCACCCGAGACGTCAACTATGGCTGAATAATCCGCTATCTACATGCCAAC

L.lagothricha TTGCCCGAGACGTAAACTACGGATGAATAATCCGCTACCTACACGCCAAC

C.libidinosus TCACCCGAGATATCAACTATGGCTGAATAATCCGCCTCCTACACGCCAAT

A.g.guariba GGCGCCTCCATATTCTTCATCTGCCTCTTCCTTCACATTGGCCGAGGCCT

A.g.clamitans GGCGCCTCCATATTCTTCATCTGCCTCTTCCTTCACATTGGCCGAGGCCT

A.sara GGCGCCTCCATATTCTTCATCTGCCTCTTCCTCCACATTGGCCGAGGCTT

A.macconneJ GGCGCCTCCATATTCTTCATCTGCCTCTTCCTCCACATTGGCCGAGGCTT

A.s.arctoidea GGCGCCTCCATATTTTTCATCTGCCTCTTCCTCCACATTGGCCGAGGCTT

A.caraya GGCGCTTCCATATTCTTCATCTGCCTATTCCTCCACATTGGCCGAGGCTT

A.belzebul GGCGCCTCCATATTCTTTATCTGCCTCTTTCTCCACATTGGCCGAGGCTT

A.pigra GGCGCCTCCATATTCTTTATCTGTCTCTTTCTCCACATTGGCCGAGGCTT

A.palliata GGCGCCTCCATATTCTTTATCTGCCTCTTTCTCCACATTGGCCGAGGCTT

L.lagothricha GGTGCTTCCATATTCTTTATTTGCCTCTTCCTACACGTCGGCCGAGGCTT

C.libidinosus GGTGCCTCCGTATTTTTTGCATGCTTATTCCTCCACATCGGCCGAGGCCT

A.g.guariba ATATTATGGATCATTCCTTTTTCTGAAGACCTGAAACATCGGTATTATCC

A.g.clamitans ATATTATGGATCATTCCTTTTTCTGAAGACCTGAAACGTCGGTATTATCC

A.sara ATATTACGGATCATTCCTTTTTCTGAAGACCTGAAACGTCGGTATTTTCC

A.macconneJ ATATTATGGATCATTCCTTTTTCTGAAGACCTGAAACGTCGGTATTATCC

A.s.arctoidea ATATTACGGATCATTCCTTTTTCTGAAGACCTGAAACGTCGGTATTATCC

A.caraya ATATTACGGGTCATTCCTTTTTCTGAAGACCTGAAACGTCGGTATTATCC

A.belzebul ATATTACGGATCATTCCTTTTTCTGAAGACCTGAAACGTCGGTATTATCC

A.pigra ATATTACGGATCATTCCTTTTTCTGGAGACCTGAAACATCGGTATTATCC

A.palliata ATATTACGGATCATTCCTTTTTCTGGAGACCTGGAACGTCGGTATTATCC

L.lagothricha ATATTATGGATCCTTCCTTTCTCTGGAGACTTGAAACGTAGGTATTATTC

C.libidinosus CTACTACGGATCCTTTCTCCTTCTAAAGACCTGAAACATCGGTACAATCC

A.g.guariba TTCTACTCACAACTATAGCCACAGCATTCATAGGCTATGTCCTCCCATGA

A.g.clamitans TTCTACTCACAACTATAGCCACAGCATTCATAGGCTATGTCCTCCCATGA

A.sara TCCTACTCACAACCATAGCTACAGCATTCATAGGCTATGTCCTCCCATGA

A.macconneJ TCCTACTCACAACCATAGCCACAGCATTCATAGGCTATGTCCTCCCATGG

A.s.arctoidea TCCTACTCACAACCATAGCCACAGCATTCATAGGCTATGTCCTCCCATGA

A.caraya TCCTACTCACAACCATAGCCACAGCATTCATAGGCTACGTCCTCCCATGA

A.belzebul TCCTACTCACAACTATAGCCACAGCATTCATAGGCTATGTCCTCCCATGA

A.pigra TCCTACTCACAACCATAGCCACAGCATTCATAGGCTATGTCCTCCCATGA

A.palliata TCCTACTCACAACCATAGCCACAGCATTCATAGGCTATGTCCTCCCATGA

L.lagothricha TACTACTTACAACCATAGCCACAGCATTCATAGGTTACGTCCTCCCATGG

C.libidinosus TACTATTAATAACAATAGCCACAGCCTTTATAGGCTACGTATTGCCGTGG

A.g.guariba GGCCAAATATCATTCTGAGGGGCCACAGTAATTACAAACCTTCTATCAGC

A.g.clamitans GGCCAAATATCATTCTGAGGGGCCACAGTAATTACAAACCTCCTATCAGC

A.sara GGCCAAATATCATTCTGAGGGGCCACAGTAATTACAAACCTTCTATCAGC

A.macconneJ GGCCAAATATCATTCTGAGGGGCCACAGTAATTACAAACCTTCTATCAGC

A.s.arctoidea GGCCAAATATCATTCTGAGGTGCCACAGTAATTACAAACCTCCTATCAGC

A.caraya GGCCAAATATCATTCTGAGGGGCCACAGTAATTACAAACCTTCTATCAGC

A.belzebul GGCCAAATATCATTCTGAGGGGCCACAGTAATTACAAATCTTCTATCAGC

A.pigra GGCCAAATATCATTCTGAGGCGCCACAGTAATTACAAACCTTCTGTCAGC

A.palliata GGCCAAATATCATTCTGAGGGGCCACAGTAATTACAAACCTTCTGTCAGC

L.lagothricha GGCCAAATATCATTCTGAGGGGCTACAGTAATCACAAATCTTCTATCAGC

C.libidinosus GGCCAAATATCATTCTGAGGGGCCACAGTTATTACAAACCTTTTATCAGC

A.g.guariba CATCCCATACATCGGATCTGACCTCGTACAATGAATCTGGGGTGGTTTCT

A.g.clamitans CATCCCATACATCGGATCTGACCTCGTACAATGAATCTGAGGTGGTTTCT

A.sara CATCCCATACATCGGATCTGACCTCGTACAATGAATCTGAGGTGGCTTCT

A.macconneJ CATCCCATACATCGGATCTGACCTCGTACAATGAATCTGAGGCGGCTTCT

A.s.arctoidea CATCCCATACATCGGATCTGACCTCGTACAATGAATCTGAGGTGGCTTCT

A.caraya CATCCCATACATCGGATCTGACCTCGTACAATGAATCTGAGGTGGGTTCT

A.belzebul CATCCCATACATCGGATCTGATCTCGTACAATGAATCTGAGGTGGTTTCT

A.pigra CATCCCATATATCGGATCTGACCTTGTACAATGAATCTGAGGTGGCTTCT

A.palliata CATTCCATATATCGGGTCTGACCTCGTACAATGAATCTGAGGTGGCTTCT

L.lagothricha CATCCCCTATATTGGGTCCAGTCTTGTAGAGTGAATCTGAGGTGGTTTCT

C.libidinosus CATCCCCTATACCGGACATGACCTTGTACAATGAATCTGAGGTGGCTTTT

A.g.guariba CAGTAGATAAAGCCACCCTCACACGATTTTTCACCTTTCACTTCATTCTA

A.g.clamitans CAGTAGATAAAGCCACCCTCACACGATTTTTCACCTTTCACTTCATTCTA

A.sara CAGTAGATAAAGCCACCCTCACACGATTTTTCACCTTTCACTTTATTCTA

A.macconneJ CAGTAGATAAAGCCACCCTCACACGATTTTTCACCTTTCACTTTATTCTA

A.s.arctoidea CAGTAGATAAAGCTACCCTCACACGATTTTTCACCTTCCACTTTATCTTA

A.caraya CAGTAGATAAAGCCACCCTTACACGATTTTTCACCTTTCACTTTATTTTA

A.belzebul CAGTAGATAAAGCCACCCTCACACGATTTTTCACCTTTCACTTTATTCTA

A.pigra CAGTAGATAAAGCCACCCTCACACGATTTTTCACCTTTCACTTTATTTTA

A.palliata CAGTAGATAAAGCCACCCTCACACGATTTTTCACCTTTCACTTTATCTTG

L.lagothricha CAGTAGACAAAGCCACCCTTACACGATTCTTTACTTTCCACTTTATCTTA

C.libidinosus CAGTGGATAAGCCCACCCTCACACGATTCTTTACCTTTCACTTTATTTTA

A.g.guariba CCCTTTATTATCGCTGCCCTAGCAACCATCCATCTCTTGTTTCTGCATGA

A.g.clamitans CCCTTTATTATCGCTGCCCTAGCAACCATCCACCTCTTGTTTCTGCATGA

A.sara CCCTTTATCATCGCTGCCCTAGCAACCATCCACCTCTTGTTTCTGCATGA

A.macconneJ CCCTTTATCATCGCTGCCCTGGCAACCATCCACCTCTTGTTTCTGCATGA

A.s.arctoidea CCCTTTATTATCGCTGCCCTAGCAACCATCCACCTCTTGTTTCTGCATGA

A.caraya CCCTTTATCATTGCTGCCCTAGCAACTATCCACCTCTTGTTTCTGCATGA

A.belzebul CCCTTTATTATCGCTGCCCTAGCAACCATTCACCTCTTGTTTCTGCATGA

A.pigra CCATTTATTATCGCTGCCCTAGCAACCATCCATCTCTTGTTTCTGCATGA

A.palliata CCATTTATTATCGCTGCCCTAGCAACCATCCACCTCTTGTTTCTGCATGA

L.lagothricha CCCTTTATTATTGCAGCCCTAGCAACTATTCACCTATTATTTCTGCATGA

C.libidinosus CCTTTCATTATCACAGCTCTAACAACCATTCACCTCTTATTTCTGCATGA

A.g.guariba AACAGGATCAAGTAACCCATCAGGAATGACATCGGACCTCGACAAAATCA

A.g.clamitans AACAGGATCAAGTAACCCATCAGGAATGACATCGGACCTTGACAAAATCA

A.sara AACTGGATCAAGTAACCCATCAGGAATAGCATCAGACCCCGATAAAATCA

A.macconneJ AACAGGATCAAGTAATCCATCAGGAGTAGCATCAGACCTCGATAAAATCA

A.s.arctoidea AACAGGATCAAGTAACCCATCAGGAATGACATCAGACCTCGACAAAATCA

A.caraya GACAGGATCAAGTAACCCATCAGGAATGGCATCAGACCTCGACAAAATCA

A.belzebul AACAGGATCAAGTAACCCATCAGGAATAACATCAGACCTCGATAAAATCA

A.pigra AACAGGATCAAGTAACCCGTCAGGAATGGCATCAGACCTCGACAAAATTA

A.palliata AACAGGATCAAGTAACCCATCAGGAGTAGCATCGGACCTCGACAAAATTA

L.lagothricha CACAGGGTCAAGTAATCCATCAGGAATAACATCAGACCCAGATAAAATCA

C.libidinosus AACAGGCTCAAATAACCCATCAGGAATAACATCCAACCCCGATAAAATTA

A.g.guariba CATTTCACCCCTACTATACAACCAAAGATATCCTAGGCCTAATTTTCCTC

A.g.clamitans CATTTCACCCCTACTATACAACCAAAGATATCCTAGGCCTAACTATTCTC

A.sara CATTTCACCCTTATTATACAACCAAAGACATCCTAGGTCTAATCATTCTC

A.macconneJ CATTTCACCCCTATTATACAATCAAAGACATCCTAGGTCTAATTATTCTC

A.s.arctoidea CATTTCACCCCTATTATACAACCAAAGACATTCTAGGCCTAATCATTCTC

A.caraya CATTTCACCCCTACTATACAACCAAAGACATCCTAGGCCTAATTATTCTC

A.belzebul CATTTCACCCCTACTATACAACCAAGGACATCCTAGGCCTAATTATTCTC

A.pigra CATTTCACCCCTACTATACAACCAAAGACATCCTAGGCTTAATTATTCTC

A.palliata CATTTCACCCCTACTATACAACCAAAGATATCCTAGGCTTAATTATTCTC

L.lagothricha CATTCCACCCCTACTATACAATCAAGGACATTTTTGGTTTAATTCTTCTT

C.libidinosus CATTCCATCCCTATTACACAACCAAAGACATTTTTGGACTAACCCTTCTT

A.g.guariba CTCCTATGTCTAACAAGCCTGACCCTATTTTCACCTGACCTTCTAACCGA

A.g.clamitans CTCCTATGTCTAATAAGCCTGACCCTATTTTCACCTGACCTTCTAACCGA

A.sara CTCCTATGCCTAATAAGCCTAACCCTATTTTCACCTGACCTTCTAACCGA

A.macconneJ CTCCTATGCCTAATAAGCCTAACCCTATTTTCACCTGACCTTCTAACTGA

A.s.arctoidea CTCCTATGCCTAGTAAGCCTAACCCTATTTTCACCTGACCTTCTAACTGA

A.caraya CTCCTATGTCTAATAAGCCTAACCCTATTTTCACCTGACCTTCTAACCGA

A.belzebul CTCCTATGTCTAATAAGCCTAACCCTATTTTCACCTGACCTTCTAACCGA

A.pigra CTTCTATGCCTAATGAGCCTAACTCTATTTTCACCCGACCTTTTAACCGA

A.palliata CTCCTATGCCTAATAAGCCTAACCCTATTTCTACCCGACCTTCTAACCGA

L.lagothricha CTCCTATGCTTAATAAACCTAACCTTATTCTCACCTGACCTCTTA-----

C.libidinosus CTCTTGCTCCTCATAAACCTAACCCTATTTACTCCTGACCTTTTAATCGA

A.g.guariba CCCAGATAATTATACACTAGCTAATCCCCTCAACACTCCACCCCA-----

A.g.clamitans CCCAGATAATTATACACTAGCTAATCCCCTCAACACTCCACCCCACATTA

A.sara CCCAGATAATTACACACTAGCTAACCCCCTCAACACCCCACCCCA-----

A.macconneJ CCCAGATAATTACACACTAGCTAATCCCCTCAACACTCCACCCCA-----

A.s.arctoidea CCCAGATAATTATACACTAGCTAACCCCCTCAACACCCCACCCCA-----

A.caraya CCCAGATAATTACACACTAGCTAACCCCCTCAACACCCCACCCCACATTA

A.belzebul CCCAGATAATTATACACTAGCTAATCCCCTCAACACCCCGCCCCACATTA

A.pigra CCCAGACAATTATACACTAGCTAATCCCCTCAACACCCCACCTCA-----

A.palliata CCCAGACAATTATACACTAGCCAACCCTCTCAACACCCCACCTCA-----

L.lagothricha --------------------------------------------------

C.libidinosus CCCAGACAACTTCACACTAGCTAACCCCCTGAATACTCCACCTCATATTA

;

end;
