# Supplementary material for: Multiple sex chromosome systems in howler monkeys (Platyrrhini, Alouatta)
Source: Comp Cytogenet. 2014 Feb 25;8(1):43–69. doi: 10.3897/CompCytogen.v8i1.6716 (PMC3978242; doi:10.3897/CompCytogen.v8i1.6716)

**Figure S (Supplementary Material)**

S: a and b). Most parsimonious trees obtained for the chromosomal partition. c). 50% majority consensus tree obtained by “bootstrap”. d) 50% majority consensus tree obtained by “bootstrap” for the molecular partition


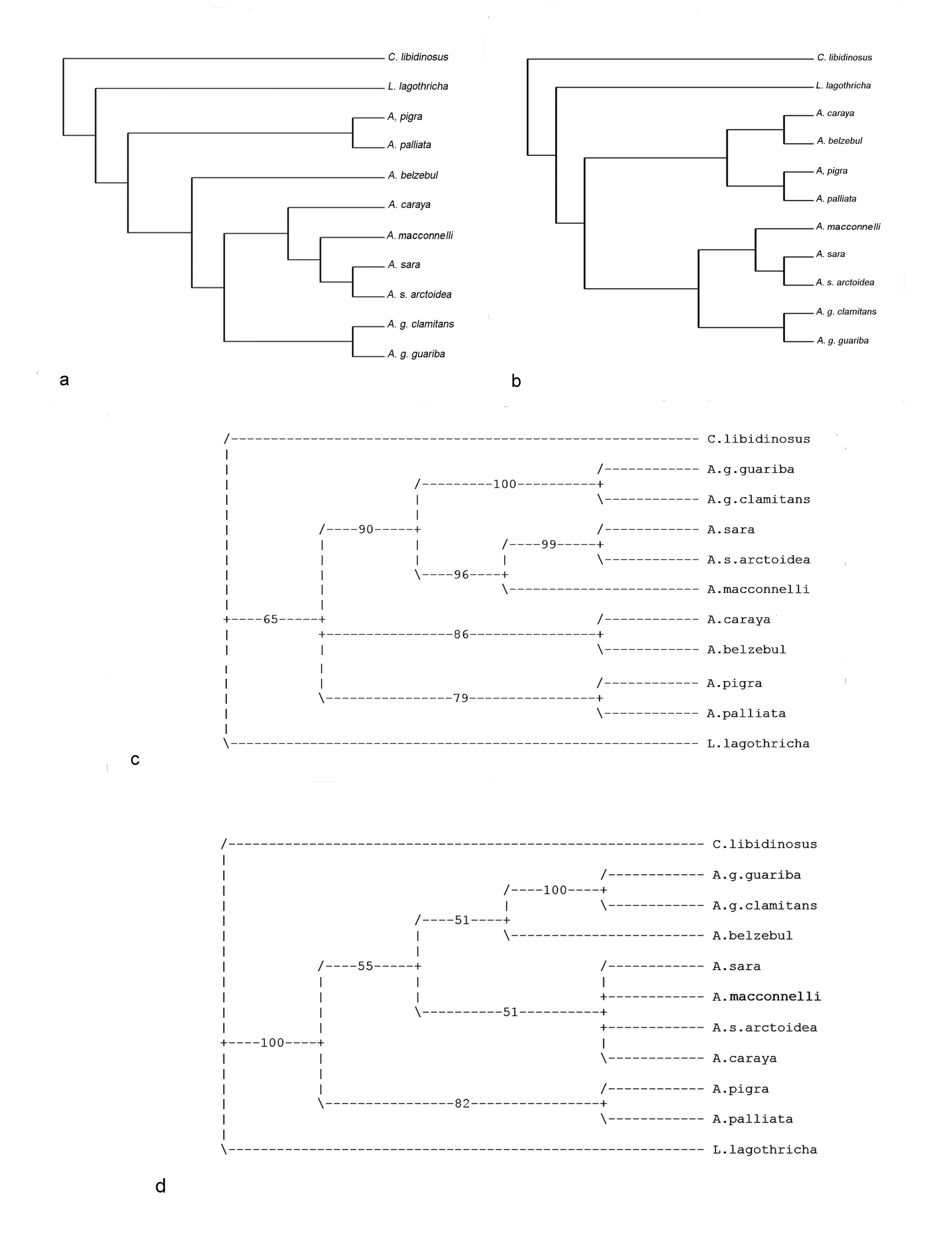

Supplement: Supplementary file 2 — Supplementary Figure S. (doi: 10.3897/CompCytogen.v8i1.6716.app2) File format: Microsoft Word file (doc). [file CompCytogen-008-043-s002.doc]
